# Research on the effect of *Rosa roxburghii* root in alleviating spleen and stomach damp–heat gastric ulcer by regulating the imbalance of oral–gut axis microbiota

**DOI:** 10.3389/fmicb.2025.1701829

**Published:** 2025-11-07

**Authors:** Jiangsong Cao, Ziyu Zhang, Xiongwei Liu, Ying Zhou, Jiaxin Li, Xinyue Wang, Chang Liu

**Affiliations:** 1 School of Pharmacy, Guizhou University of Traditional Chinese Medicine, Guiyang, Guizhou, China; 2 Guizhou Key Laboratory of Modern Traditional Chinese Medicine Creation, Guiyang, Guizhou, China

**Keywords:** spleen and stomach, spleen stomach damp-heat gastric ulcer, *Rosa roxburghii* root, oral-gut microbial axis, rothia, corynebacterium, streptococcus, romboutsia

## Abstract

**Background:**

*Rosa roxburghii* root (RT), a medicinal herb traditionally utilized by ethnic minorities in Guizhou Province, has demonstrated potential in managing gastrointestinal disorders. Nonetheless, its effectiveness in treating gastric ulcers (GU) accompanied by spleen–stomach damp–heat syndrome, especially through mechanisms that involve interactions with oral–gut microbiota, remains to be elucidated.

**Methods:**

A rat model of GU with damp–heat syndrome was established. The rats were treated with various doses of RT, and gastric mucosal injury was assessed through ulcer index calculation and histopathological examination. Additionally, the levels of immunoglobulin 6 (IL-6), tumor necrosis factor alpha (TNF-*α*), nitric oxide (NO), inducible nitric oxide synthase (iNOS), motilin (MTL), prostaglandin E_2_ (PGE_2_), and malondialdehyde (MDA) were measured. A 16S ribosomal RNA (rRNA) sequencing was conducted on samples of tongue coating and intestinal contents to analyze the microbial composition and changes.

**Results and discussion:**

Compared to the control (CON) group, the gastric ulcer (GU) group exhibited significant pathological alterations in the gastric mucosa. The levels of IL-6, TNF-*α*, and MDA were significantly elevated (*p <* 0.01), whereas the levels of NO, iNOS, MTL, and PGE_2_ showed a notable reduction (*p <* 0.01). Compared to the GU group, the RT’s high-dose (RTH) groups exhibited statistically significant improvements in the ulcer index, reduced levels of TNF-*α*, IL-6, MDA, and increased levels of NO, MTL, iNOS, and PGE_2_ (*p <* 0.05). Moreover, RT reversed oral–gut microbial dysbiosis, increasing the relative abundance of oral bacteria *Muribacter* and *Corynebacterium*, as well as intestinal bacteria *Lactobacillus*, *Romboutsia*, and *Limosilactobacillus*, while decreasing the relative abundance of oral bacteria *Rodentibacte*r, *Rothia*, and *Streptococcus*, and intestinal bacteria *Ligilactobacillus* and *Desulfovibrio*. Both oral and gut bacteria are closely associated with clinical inflammatory factors in GU. Following ulcer onset, decreased levels of NO, iNOS, PGE_2_, and MTL, alongside increased levels of TNF-*α*, IL-6, and MDA, directly induce a reduction in the abundance of bacteria, including *Rothia*, *Streptococcus*, *Corynebacterium*, *Globicatella*, *Romboutsia*, and *Lactobacillus*, with this effect being more pronounced in the oral cavity. However, treatment with RT may potentially increase the abundance of these bacteria within the intestine, which could directly regulate gastric ulcer-related inflammatory factor levels and ameliorate clinical symptoms. *R. roxburghii* root has therapeutic effects against the progression of gastric ulcers by promoting mucosal repair and suppressing the release of inflammatory mediators.

## 1 Introduction

Gastric ulcer (GU), a prevalent subtype of peptic ulcer disease, manifests clinically with symptoms such as hunger discomfort, postprandial epigastric pain, bloating, belching, and acid regurgitation. In severe cases, patients may present with melena, hematemesis (Luo et al., 2025), and potential complications including perforation or malignant transformation (Wang et al., 2025). Epidemiological studies indicated that its global prevalence ranges from 5 to 10% (Yang et al., 2024), significantly impairing quality of life and escalating healthcare burdens. Current therapeutic strategies predominantly employ histamine H_2_-receptor antagonists and proton pump inhibitors (e.g., omeprazole), yet these approaches face clinical limitations, including frequent adverse effects and high recurrence rates.

Gastric ulcer is categorized as “epigastric pain” in Traditional Chinese Medicine (TCM), with its pathogenesis often attributed to multiple factors, including the six exogenous pathogenic factors, emotional dysregulation, dietary irregularities, and constitutional susceptibility. The fundamental pathological mechanism involves a deficiency in the spleen and stomach, resulting in impaired digestive and transport functions. This leads to qi stagnation in the middle jiao, insufficient nutrient production, and malnourishment of the gastric collaterals, ultimately manifesting as pain due to tissue deficiency. Clinically, the spleen–stomach dampness-heat syndrome is the predominant syndrome differentiation type (Fu et al., 2022). In cases of dampness-heat syndrome gastric ulcers, the retention of “heat-toxicity” in the spleen–stomach combined with prolonged damp–heat accumulation causes tissue erosion. Therapeutic strategies focusing on heat-clearing, dampness-resolving, qi-regulating, and stomach-harmonizing have demonstrated efficacy in alleviating clinical symptoms (Cao et al., 2025). The Miao ethnic medicine “Jiaohuan Theory” interprets symptoms such as epigastric pain, acid reflux, hiccups, and vomiting as manifestations of upper jiao disharmony. According to this theory, pathological changes stem from “jiaohuan” dysfunction, thereby necessitating therapeutic interventions to reinstate “jiaohuan” coordination. The Miao medical principle of “fortifying the stomach to harmonize jiaohuan” guides pharmacological interventions, emphasizing the restoration of visceral.

TCM represents a conventional therapeutic approach for GU through multi-target and multi-pathway mechanisms. Both single herbs and compound formulations can modulate gastric acid secretion and promote ulcer astringency (Gong et al., 2024), demonstrating broad-spectrum bioactivity, favorable therapeutic efficacy, and reliable safety profiles (Wang et al., 2024). For instance, roots of *Bupleuri radix*, *Mori folium*, *Inulae flos*, and *Crataegi fructus* have shown gastric mucosal protective effects against various *in vivo* ulcer models (Chen et al., 2022). Furthermore, medicinal materials such as *Coptidis rhizoma*, *Atractylodis macrocephalae rhizoma*, and *Atractylodes lancea* exhibit inhibitory effects against *Helicobacter pylori* infection while reducing mucosal lesion areas and neutrophil infiltration to facilitate gastric mucosal healing (Wang et al., 2015; Li et al., 2022; Qu et al., 2022). However, the mechanistic underpinnings of most TCM interventions for GU remain incompletely elucidated, necessitating systematic investigations to identify optimal herbal candidates for clarifying their anti-ulcer mechanisms.

The *Rosa roxburghii* root (RT), a member of the *Rosaceae* family, is characterized by its cooling properties and association with the “heat channel” in Miao ethnomedicine. Traditionally, it is believed to affect four anatomical systems: the belly, lungs, body, and cavities, by strengthening the stomach, promoting digestion, and replenishing deficiencies (Guizhou Provincial Drug Administration, 2003). In Guizhou folk medicine, it is frequently decocted into concentrated solutions and used as a daily tea substitute, or it is soaked in liquor with *Cibotium barometz* to create medicinal wine. Additionally, this plant is commonly utilized to treat digestive disorders such as indigestion, abdominal distension, diarrhea, dysentery, and enteritis (Yan et al., 2024), and is classified as a stomach-strengthening and harmonizing agent. Recent pharmacological studies have indicated that the anti-inflammatory properties of RT are mainly due to its bioactive components, including triterpenoids, ellagic acids, flavonoids, and oligosaccharides (Liang et al., 2022). In a chronic ulcer rat model, RT extract demonstrated ulcer-healing properties by inhibiting lipid peroxidation in the glandular gastric mucosa, enhancing mucosal antioxidant capacity, and alleviating oxygen free radical-induced damage (Chen et al., 2001). Although RT exhibits unique therapeutic benefits in the clinical management of gastric ulcers, the underlying mechanisms are not yet fully understood.

The oral–gut microbiota axis is a central focus in gastric ulcer research, with its dynamic interaction with herbal medicines playing a crucial role in maintaining host homeostasis. The occurrence of gastrointestinal disorders disrupts the harmonious balance of the spleen’s ascending and stomach’s descending functions, leading to destabilization of the oral environment. Pathogenic qi may descend from the oral cavity into the esophagus, stomach, and intestines through the digestive tract, thereby inducing gut microbiota dysbiosis and subsequent gastrointestinal pathologies (Yu et al., 2023; Shen et al., 2025). Tongue coating, formed by the upward steaming of food essence through the spleen and stomach qi, reflects disturbances in the tongue coating microbiota when splenic-gastric dysfunction occurs. The tongue coating microbiota constitutes a critical component of both the gastrointestinal microecosystem and the oral microbial community. While distinct from the gut microbiota, it interacts bidirectionally with intestinal microbial populations (Wu et al., 2025). Under pathological conditions, a close microbial interplay exists between the oral cavity and gut: oral microbes may descend to the intestines, exacerbating gastrointestinal diseases, while gut microbes can ascend to the oral cavity, altering its microbial composition (Zhang et al., 2024). Gastric ulcers, following mucosal damage, further disrupt gut microbiota equilibrium and modify microbial profiles (Li et al., 2025). Cross-talk between the tongue coating microbiota and gut microbiota contributes to distinct tongue manifestation characteristics and influences the progression and prognosis of gastric ulcers (Liu et al., 2023).

In this study, RT was extracted using the aqueous extraction method, a Traditional TCM technique known for its efficiency, cost-effectiveness, and ability to preserve bioactive components (Ma, 2020). Additionally, an improved animal model was developed to replicate spleen–stomach damp–heat syndrome gastric ulcer by exposing rats to a high-temperature and high-humidity environment, high-fat diet, and 56% ethanol solution (Wang et al., 2024). The therapeutic effects and molecular mechanisms of RT extract were evaluated by analyzing biochemical indicators and assessing the composition and structural alterations of oral–gut microbiota in rats. Therefore, this study aimed to investigate the therapeutic effects of RT on GU with damp–heat syndrome and to explore whether its mechanism is related to the regulation of oral–gut microbiota and inflammatory responses.

## 2 Materials and methods

### 2.1 Animals

A total of 60 Wistar rats, each weighing between 180 and 220 g and of specific-pathogen-free (SPF grade), were utilized in this study. They were procured from Changsha Tianqin Biotechnology Co., Ltd., bearing the production license number SCXK (Xiang) 2022–0011. The rats were accommodated in the animal experimental center at Guizhou University of Traditional Chinese Medicine, where they had unrestricted access to both food and water. Following a week of acclimatization with their feed, the experiment was initiated. The animal experiment protocol was examined and given ethical approval by the Ethics Committee of Guizhou University of Traditional Chinese Medicine (approval number: 2024048).

### 2.2 Reagents

The immunoglobulin 6 (IL-6) kit (ZC-36404), tumor necrosis factor alpha (TNF-*α*) kit (ZC-37624), inducible nitric oxide synthase (iNOS) kit (ZC-37563), motilin (MTL) kit (ZC-37312), prostaglandin E_2_ (PGE_2_) kit (ZC-37100) were purchased from Shanghai ZCIBIO Technology Co., Ltd. (Shanghai, China); malondialdehyde (MDA) kit (A003-1-1) and NO kit were purchased from Nanjing Jiancheng Bioengineering Research Institute (Nanjing, China); Omeprazole enteric-coated tablets was purchased from Shandong New Times Pharmaceutical Co., Ltd. (Shandong, China); Acetonitrile (ACN) and 98.0% methanol were purchased from Fisher Chemical (USA); 98.0% formic acid (FA) was purchased from Sigma–Aldrich (Germany); The Lard, sugar, honey, and Erguotou are sold in the market.

### 2.3 Sample preparation and identification

#### 2.3.1 Sample preparation

The *R. roxburghii* root (RT) is harvested from the Huaxi District in Guiyang City, Guizhou Province. It was identified by Professor Chang Liu from the Guizhou University of Traditional Chinese Medicine as belonging to the Rosaceae family. The specimen is archived at the School of Pharmacy, Guizhou University of Traditional Chinese Medicine (specimen number: CL20240318). After drying and grinding the root into powder, it is soaked in distilled water for 20 min, then boiled vigorously for 30 min and simmered gently for 1 h. This process is repeated once, and the two decoctions are combined. The concentrated medicinal liquid is adjusted to a concentration of 1 g/mL and stored in a refrigerator at 4°C.

#### 2.3.2 Chromatography and mass spectrometry conditions

A 100 mg aliquot of freeze-dried RT extract was placed in a screw-capped conical flask, to which 1,000 μL of methanol was added. The mixture was shaken for 10 min, centrifuged at 12,000 rpm for 10 min, and the supernatant was taken for analysis. Due to the absence of identification parameters for RT in both the Chinese Pharmacopoeia and Guizhou provincial standards, we established its identification based on reported methods for content determination and fingerprint profiling (Li, 2018), using the four compounds ellagic acid, gallic acid, trans-ferulic acid, and rutin.

The chromatographic separation was performed on an Acquity™ Premier HSS T3 C_18_ column (100 mm × 2.1 mm, 1.8 μm) using a mobile phase consisting of 0.1% formic acid in water (A) and 0.1% formic acid in acetonitrile (B). The gradient elution program was as follows: from 0 to 1 min, 2% B; from 1 to 9 min, 2–50% B; from 9 to 13.5 min, 50–98% B; and from 13.5 to 20 min, 98–2% B. The flow rate was set at 0.25 mL/min with an injection volume of 10 μL, and the column temperature was maintained at 45°C. The mass spectrometry (MS) parameters were set as follows: mass spectrometry 1 (MS1) resolution, 70,000; AGC target, 1e6; maximum injection time, 50 ms; scan range, *m/z* 150–1,500; MS2 resolution, 17,500; AGC target, 1e5; maximum injection time, 50 ms; TopN, 10; with normalized collision energy (stepped NCE) at 10, 30, and 55.

### 2.4 Animal experimental design

**Figure 1:**
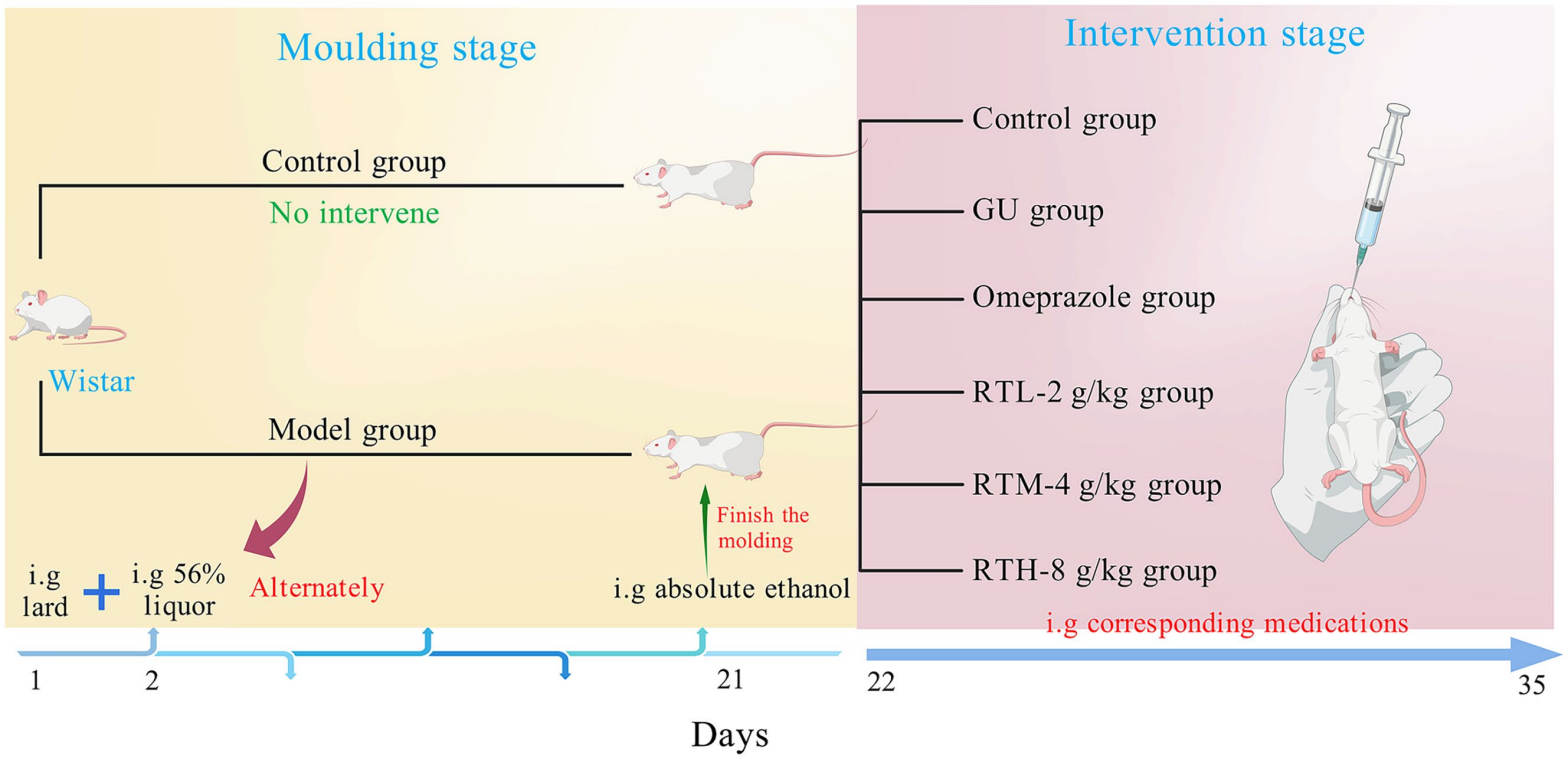
Design of animal experiments.

The 60 Wistar rats (male-to-female ratio of 1:1) were divided into 6 groups, each consisting of 10 rats: the control group (CON), the gastric ulcer (GU) group, the omeprazole group (OP), and the RT’s low-dose (RTL, 2 g/kg) group, medium-dose (RTM, 4 g/kg) group, high-dose (RTH, 8 g/kg) group. Apart from the control group, which did not undergo any intervention, all other animals were used to establish a spleen–stomach damp–heat syndrome gastric ulcer model (Figure 1). They were kept in a humid environment, fed every other day, given free access to drinking water, and started on a mixed drink of 10% honey and 10% sugar from day 5. From the 5th day, each rat was administered 10 g/kg of pig oil every other day and 1 mL of 56% alcohol every other day until the 20th day. On the 20th day of modeling, except for the control group, the other groups were given intragastric anhydrous ethanol at a dose of 1 mL/200 g, and fasted for 24 h before the administration of anhydrous ethanol. Throughout the entire modeling process, the rats exhibited varying degrees of symptoms, including yellow-stained fur, yellow urine, constipation, black stools, and a thick yellow tongue coating. At 3 h post-modeling, one rat was randomly selected from each group and anesthetized via intraperitoneal injection of 2% pentobarbital sodium. The entire stomach was excised, opened along the greater curvature, and flattened for observation. The presence of hyperemia, erythema, and ulcerative lesions, along with ulcerative changes observed in gastric antral tissue sections under light microscopy after Hematoxylin and Eosin (H&E) staining, confirmed the successful model establishment (Hossen et al., 2018).

According to the record in the Folk Herbs of Guiyang, RT is decocted into a thick liquid, taken three times a day for 1 week to treat chronic gastritis and stomach pain, and the dosage of RT is determined by combining literature reports and the dose translation coefficient per kilogram body weight of animals and humans. The control group was given 0.9% normal saline of the same volume; according to the conversion of adult dose and rat equivalent dose, the high, middle, and low groups of RT water decoction were given intragastrically at the doses of 8, 4, and 2 g/(kg·day), respectively. At the same time, omeprazole was converted from a daily dose of 40 mg for adults to a daily dose of 0.4 mg/100 g for rats, and the omeprazole group received 1 mL/100 g intragastrically once a day for 14 days. After the last administration, the rats were fasted for 12 h, anesthetized with 2% pentobarbital sodium, and blood was collected through the abdominal aorta of the rats. After blood collection, the rats were euthanized in accordance with the American Veterinary Medical Association (AVMA) guidelines for the Euthanasia of Animals (2020), and the method of euthanasia was carried out using the CO_2_ asphyxiation method. The whole stomach was extracted, cut along the greater gastric curvature, and the stomach contents were rinsed with 0.9% sodium chloride.

### 2.5 Detection of inflammatory factors in serum and gastric tissue

Following blood collection via abdominal aorta puncture, the serum was incubated at room temperature for 2 h, then centrifuged at 3,500 rpm for 15 min. The serum levels of NO, IL-6, and TNF-*α* inflammatory factors were subsequently detected. The remaining blood was removed from the stomach tissues, which were then added to PBS at a mass-to-volume ratio of 1:9. The tissue homogenate was ground on ice and centrifuged at 10,000 rpm at 4°C for 10 min. The contents of MDA, iNOS, MTL, and PGE_2_ in the stomach tissues were detected using supernatants, following the instructions provided with the ELISA kit.

### 2.6 Gastric mucosal injury ulcer index and gastric histopathological changes

The gastric mucosal injury ulcer index in rats was evaluated and graded using the Guth index scoring method (Song et al., 2019). Lesions with a length of ≤1 mm were scored as 1 point, those from >1 to 2 mm as 2 points, from >2 to 3 mm as 3 points, and from >3 to 4 mm as 4 points. When the lesion width exceeded 2 mm, the score was doubled. The ulcer inhibition rate was calculated using the formula: (model group ulcer index - treatment group ulcer index)/model group ulcer index × 100%. Gastric tissues were immersed in 4% formalin, dehydrated through a graded series of ethanol, and finally embedded in paraffin. The paraffin-embedded stomach block was securely fixed to the specimen holder to ensure complete exposure of the stomach section. Subsequently, 4 μm thick sections were cut and mounted with neutral gum. Microscopic examination was conducted to observe tissue morphology and structural changes in the stomach.

### 2.7 Tongue coating and intestinal flora collection in rats

The rats were anesthetized, and the tongue coating was scraped from back to front more than five times with moderate force using a sterile swab to collect the tongue coating contents. The swab head was then cut off with sterile scissors and placed in a sterile, frozen tube to obtain tongue coating samples. Subsequently, the abdominal cavity of the rat was opened to locate the ileocecal valve, and intestinal tissues from the end of the ileum and below were collected. The intestinal contents were squeezed into a sterile, frozen tube at the end of the ileum using sterile tweezers to obtain intestinal contents samples. The samples were immediately frozen in liquid nitrogen and transferred to −80°C for storage.

### 2.8 Total DNA extraction and sequencing of tongue coating and intestinal contents samples

Coated tongue and intestinal canal contents of rats were selected in the CON group, GU group, OP group, and RTH group, with five rats in each group. The five selected samples were randomly chosen and representative within the group (with balanced male-to-female distribution). DNA was extracted from coated tongue and intestinal contents using the QIAamp Fast DNA Stool Mini Kit according to the kit instructions, and the quality and quantity of the extracted DNA were determined and evidenced. Quality control of the raw sequencing data involved filtering out low-quality reads and trimming lengths, which resulted in high-quality sequences. The quality assessment metrics for the sequencing data are detailed in Supplementary Tables S1, S2. The genomic DNA template was amplified using specific primers with barcodes, synthesized based on the full-length primer sequence, targeting the V3–V4 region. The amplified products were then purified, evidenced, and normalized to create a sequencing library (SMRTbell, USA). Following library construction, it underwent quality control. Upon passing quality control, the library was sequenced using the PacBio Sequel. This experiment was conducted by Beijing Biotechnology Co., Ltd (Beijing, China). This data can be found with the accession number PRJNA1337212.

### 2.9 Sequencing data processing and species taxonomy

After sequencing with the PacBio Sequel, the lima software (version 1.7.0) is utilized to identify Circular Consensus Sequences (CCS) by barcode, thereby obtaining the original Raw-CCS sequence data. Subsequently, the sequence is processed using the cutadapt software (version 1.9.1) to identify and remove contaminants and filter by length, resulting in the Clean-CCS sequence. The Effective CCS sequence is then acquired by eliminating chimera sequences with the UCHIME software (version 4.2). Cluster analysis of the Effective CCS is conducted using Uparse software, which clusters sequences into Operational Taxonomic Units (OTUs) with a 97% identity threshold. The classification of species encompasses seven levels: kingdom, phylum, class, order, family, genus, and species.

### 2.10 Alpha diversity and partial least squares discriminant analysis

Alpha diversity analysis reflects the abundance and diversity of the gut microbiome. The Qiime software (version 1.9.1) was utilized for the Alpha diversity analysis of the samples. Mothur software was employed to calculate the parameters of the Chao1, Shannon, Simpson, and Ace indices, which characterize their diversity. Subsequently, the Rarefaction Curve was drawn. Utilizing the abundance information, the Unique Fraction (UniFrac) distance was computed using the Qiime software (version 1.9.1). The closer the distance between two samples, the more similar their composition, indicating whether there are significant microbial community differences between the samples. Partial least squares discriminant analysis (PLS-DA) was conducted on the tongue coating and gut microbes of rats in each group, and PLS-DA maps were generated using R software (version 3.1.1) and mixOmics software (version 6.3.2).

### 2.11 LEfSe analysis and correlation analysis

The linear discriminant analysis effect size (LEfSe) was utilized, with the default linear discriminant analysis (LDA) score threshold set to 4. At each classification level, the composition and structure of tongue coating and intestinal flora were analyzed, and various bacterial genera were identified. Spearman’s rank correlation analysis was conducted on the tongue coating and intestinal flora, and a correlation network was constructed using data with a correlation coefficient greater than 0.1 and a *p*-value less than 0.05. Based on the abundance and variation profiles of each species across different samples, Spearman rank correlation analysis was performed to construct a correlation network using data that met the criteria of a correlation coefficient greater than 0.1 and a *p*-value less than 0.05. Analysis of the network diagram revealed species co-occurrence relationships in environmental samples, offering insights into microbial interactions and key ecological patterns under similar environmental conditions. These findings further elucidate the underlying mechanisms contributing to phenotypic variations observed among different samples.

## 3 Results

### 3.1 Component identification in *Rosa roxburghii* root

**Figure 2:**
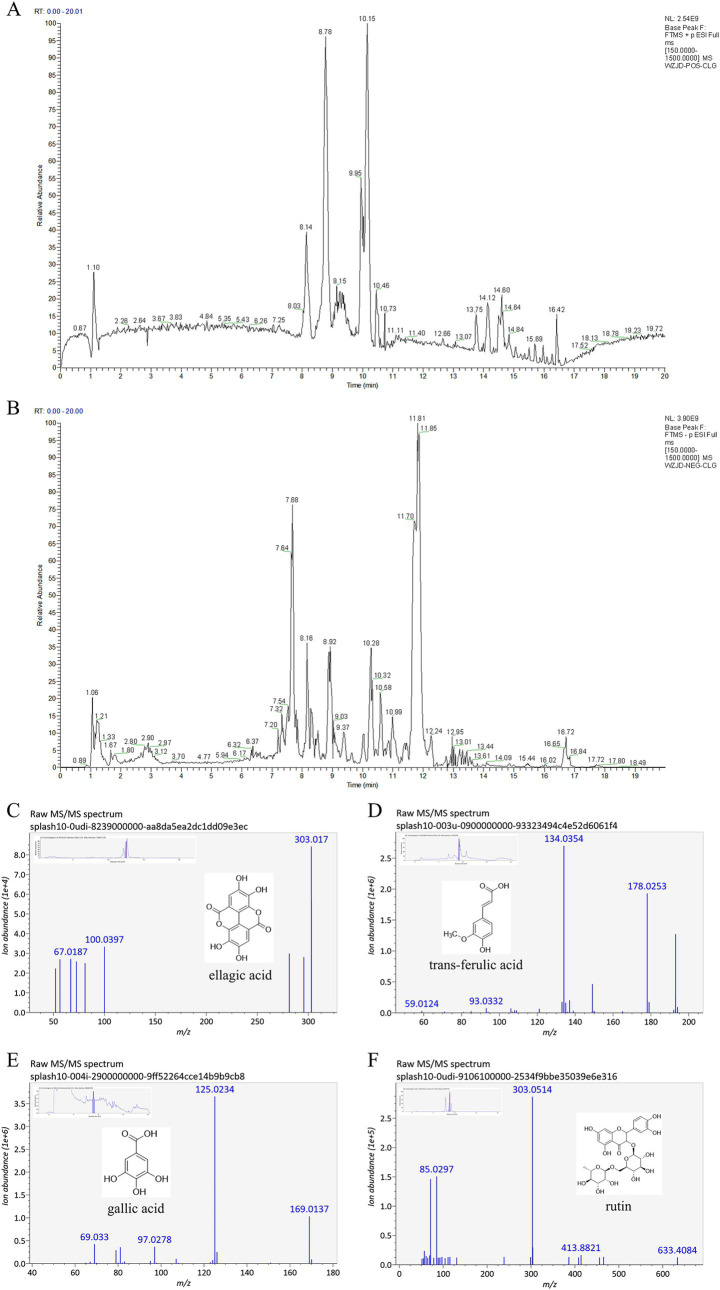
Total ion chromatogram (TIC) and mass spectral data of *Rosa roxburghii* root extracts. **(A)** Base peak chromatogram in positive ion mode; **(B)** Base peak chromatogram in negative ion mode; **(C)** MS/MS spectra and XIC of ellagic acid; **(D)** MS/MS spectra and XIC of trans-ferulic acid; **(E)** MS/MS spectra and XIC of gallic acid; **(F)** MS/MS spectra and XIC of rutin.

The total ion chromatograms (TIC) of reverse transcription (RT) extracts in positive and negative ion modes, obtained through liquid chromatography–tandem mass spectrometry (LC–MS/MS), are presented in Figures 2A,B. The MS/MS data underwent preprocessing using data-independent MS/MS deconvolution for comprehensive metabolome analysis, employing the MS-DIAL software version 4.70. Peak features were matched against three spectral libraries: assBank, ReSpect, and GNPS (with a combined total of 14,951 records), by comparing retention times, *m/z* values, and MS/MS fragmentation patterns. This process tentatively identified 56 compounds, comprising 7 phenolics, 11 flavonoids, 1 amino acid, 4 saccharides, 7 fatty acids, 8 organic acids, 3 terpenoids, 2 alkaloids, 4 coumarins, and 9 others. Phenolics and flavonoids were the predominant chemical classes identified, with the mass spectral information and compound names detailed in Supplementary Table S3. Extracted ion chromatograms (XIC) and MS/MS spectra of four representative compounds are illustrated in Figures 2C–F. Four target compounds were successfully detected in the RT extracts, with particularly high content of ellagic acid and gallic acid, confirming that the botanical material met the quality specifications for this study.

### 3.2 Therapeutic efficacy evaluation of *Rosa roxburghii* root in rats with spleen–stomach damp–heat syndrome gastric ulcer

#### 3.2.1 Effects on gastric histomorphology in rats

**Figure 3:**
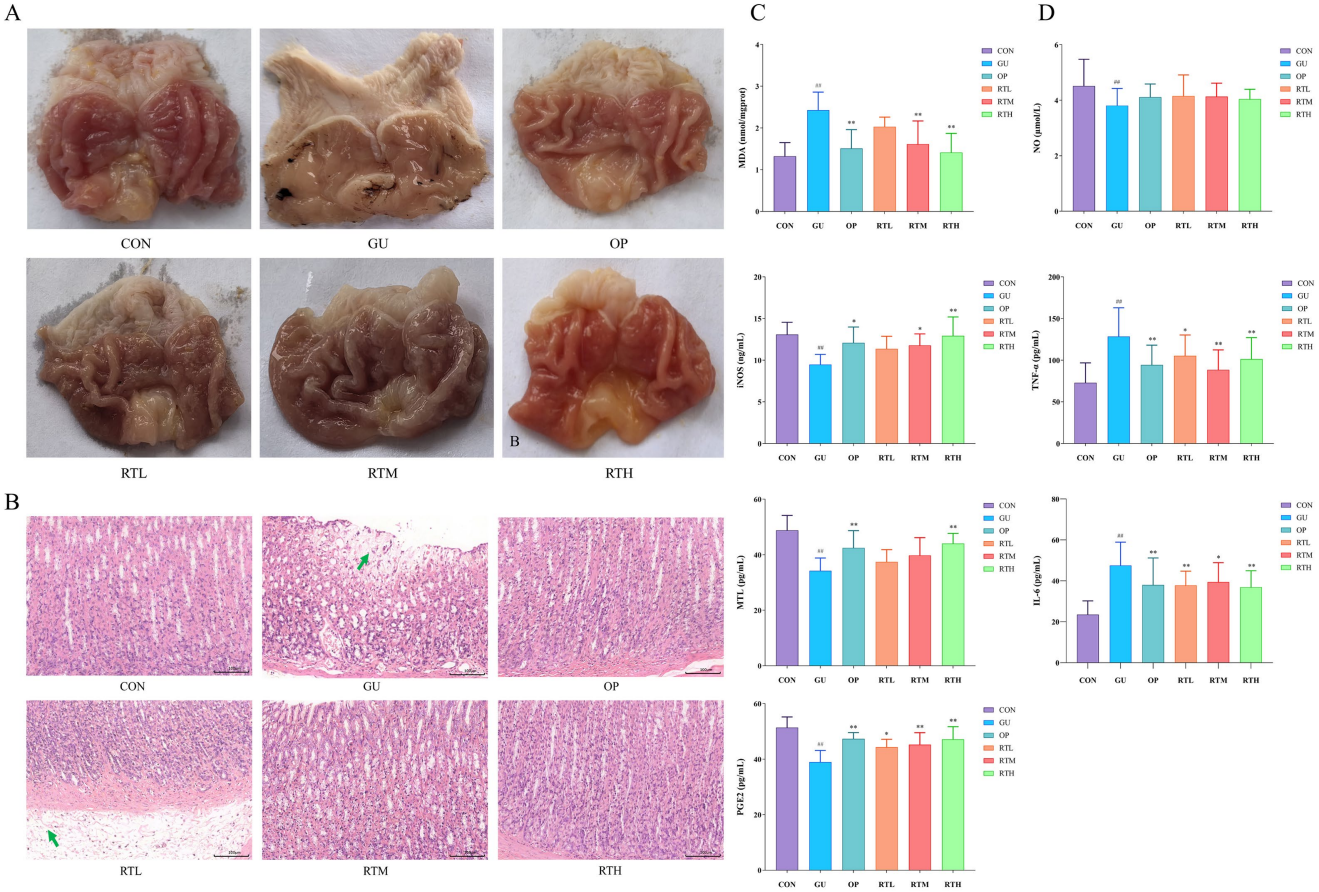
Therapeutic evaluation of *Rosa roxburghii* root in spleen–stomach damp–heat syndrome gastric ulcer rats. **(A)** Morphological changes in gastric tissue; **(B)** Histopathological alterations in gastric tissue (HE, 200×); **(C)** Changes in MDA, iNOS, MTL, and PGE_2_ levels in gastric mucosa; **(D)** Variations in serum NO, TNF-*α*, and IL-6 concentrations. Compared with the CON group: ^#^*p <* 0.05, ^##^*p <* 0.01; compared with the GU group: ^*^p < 0.05, ^**^p < 0.01.

In the control (CON) group, the rats displayed an intact gastric mucosa with reddish-pink coloration and regular rugae. Compared with the CON group, the gastric ulcer (GU) group exhibited extensive dark-red hemorrhagic lesions, ulceration, edema, and a significant increase in the gastric ulcer index (Figure 3A, Table 1; *p <* 0.01), confirming successful model establishment. In contrast, all treatment groups showed a markedly reduced area of gastric tissue damage, improved hemorrhage symptoms, and a significantly decreased gastric ulcer index compared to the GU group (Figure 3A; Table 1; *p <* 0.05, *p <* 0.01). Notably, no statistically significant difference in the rate of gastric ulcer inhibition was observed between the RTH and OP groups (Figure 3A; Table 1; *p* > 0.05).

**Table 1:** Effects of *Rosa roxburghii* root on gastric ulcer index and ulcer inhibition rate in rats with spleen–stomach damp–heat syndrome gastric ulcer.

Group	Dosage	Gastric ulcer index	Ulcer inhibition rate (%)
CON	–	–	100
GU	–	25.80 ± 14.98^##^	0
OP	4 mg/kg	6.89 ± 2.93^**^	73.30 ± 11.37^**^
RTL	2 g/kg	16.89 ± 5.49^*^	35.54 ± 21.27^**^
RTM	4 g/kg	9.43 ± 3.21^**^	63.46 ± 12.43^**^
RTH	8 g/kg	6.86 ± 3.58^**^	73.42 ± 13.87^**^

Compared with the CON group: ^#^*p <* 0.05 ^##^*p <* 0.01, compared with the GU group: ^*^p < 0.05, ^**^p < 0.01.

#### 3.2.2 Effects on gastric histopathological changes in rats

In the CON group, no significant pathological changes were observed in the gastric tissues. In contrast, the GU group exhibited necrosis of the gastric mucosa, a disordered cellular arrangement, structural blurring, submucosal edema with loosely arranged cells, and inflammatory cell infiltration. The RTL group showed submucosal edema, loosely arranged cells, and minor infiltration of inflammatory cells. Notably, the OP, RTM, and RTH groups displayed a restored gastric mucosa with no significant vascular dilation or inflammatory infiltration. All treatment groups exhibited an improvement in pathological symptoms compared to the GU group (Figure 3B).

#### 3.2.3 Effects on inflammatory and gastric mucosa repair factors in rats

Compared to the CON group, the GU group showed a significant increase in MDA levels (*p <* 0.01) and notable decreases in iNOS, MTL, and PGE_2_ levels (*p <* 0.01) in gastric mucosal tissue (Figure 3C). In contrast, both the OP and RTH groups demonstrated a significant reduction in MDA levels (*p <* 0.01) and an increase in iNOS, MTL, and PGE_2_ levels compared to the GU group (*p <* 0.01). The expression of iNOS is a dynamic process, typically rapidly induced during the early stages of inflammation, peaking at the height of the inflammatory response, and then potentially declining due to negative feedback regulation or tissue damage. Serum analysis revealed that the GU group had significantly elevated levels of IL-6 and TNF-*α* (*p <* 0.01), but reduced levels of NO (*p <* 0.01) compared to the CON group (Figure 3D). All treatment groups exhibited significant decreases in serum IL-6 and TNF-α levels in comparison to the GU group (*p <* 0.05, *p <* 0.01). Although there was an upward trend in serum NO levels within the treatment groups, the increase did not achieve statistical significance (*p* > 0.05). Therefore, following RT treatment, the levels of pro-inflammatory factors TNF-α and IL-6, as well as oxidative stress markers MDA, decreased rapidly. Concurrently, the level of the mucosal protective factor PGE_2_ recovered. This favorable microenvironment facilitated the repair at the histological level.

### 3.3 Analysis of oral (tongue coating) microbiota

#### 3.3.1 Identification and taxonomic distribution of oral (tongue coating) microbiota

**Figure 4:**
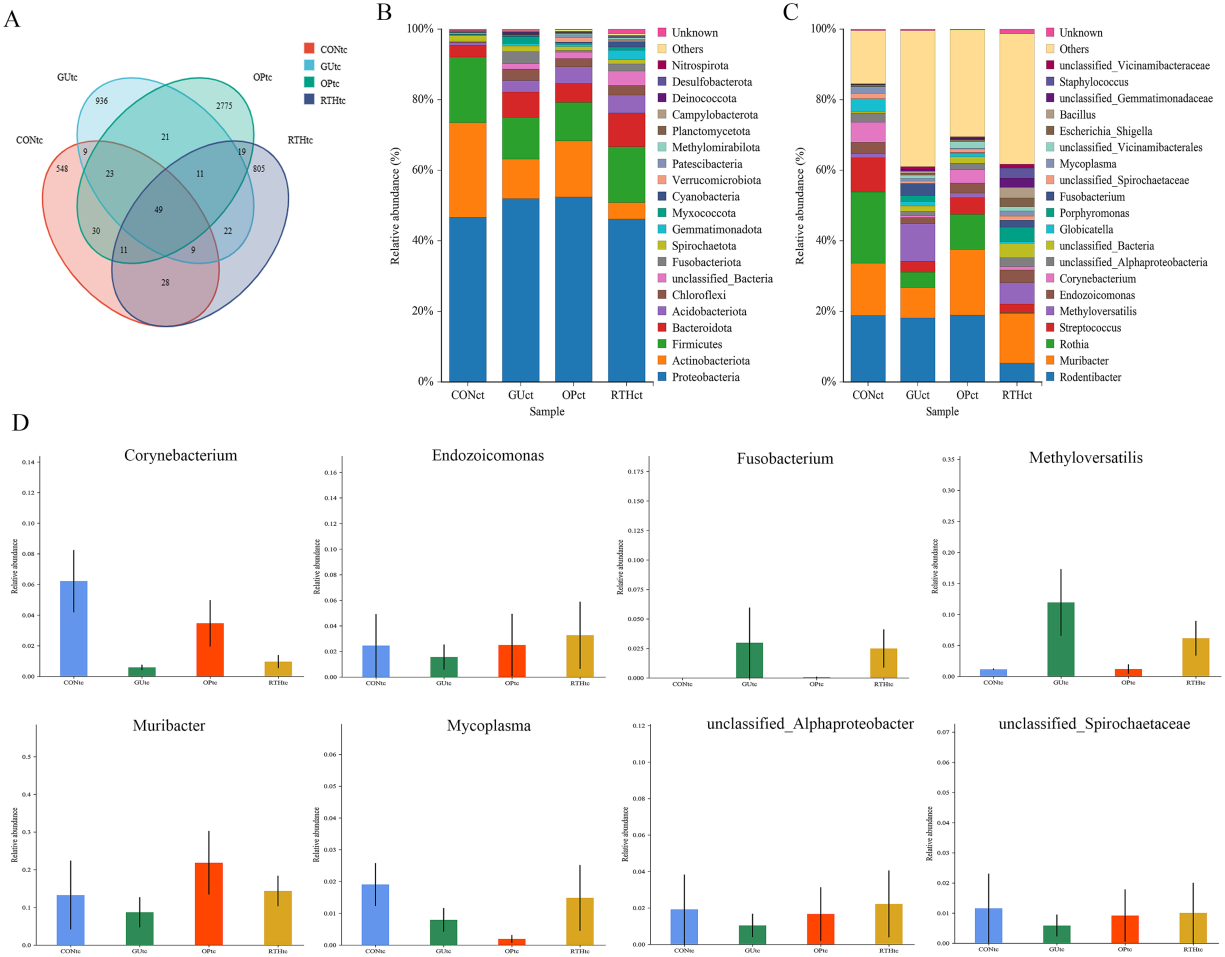
Identification and taxonomic distribution analysis of oral (tongue coating) microbiota. **(A)** Venn diagram of OTU clustering overlap; **(B)** Phylum-level relative abundance bar plot; **(C)** Genus-level relative abundance bar plot; **(D)** ANOVA of Genus-level species abundance.

To investigate the impact of RT on the structure of tongue coating flora in rats with gastric ulcers, tongue coating samples from the CON, GU, OP, and RTH groups were amplified using 16S ribosomal RNA (rRNA) DNA sequencing. A total of 1,354,258 paired reads were obtained; after quality control and splicing, 1,232,536 Clean Reads were generated, with an average of 61,627 Clean Reads per sample. Based on 97% similarity, a total of 5,296 OTUs were obtained from the 20 tongue coating samples. A Venn diagram illustrates the typical and unique flora among these samples, identifying the shared microorganisms across different environments. The CON tongue coating (CONtc) group had 707 OTUs, the GU tongue coating (GUtc) group had 1,080 OTUs, the OP tongue coating (OPtc) group had 2,929 OTUs, and the RTH tongue coating (RTHtc) group had 954 OTUs. Among these, 90 OTUs were shared between the GUtc and CONtc groups, and 91 OTUs were shared between the GUtc and RTHtc groups (Figure 4A).

Significant differences were observed in the distribution of microbial community structure and relative abundance after the administration of RT. The OTU results were applied for species annotation, and at the Phylum level, compared with the CONtc group, the relative abundance of *Proteobacteria*, *Bacteroidota*, *Acidobacteriota*, *Chloroflexi*, and *Fusobacteriota* increased. In contrast, the relative abundance of *Actinobacteriota* and *Firmicutes* decreased in the GUtc group. Compared with the GUtc group, the relative abundance of *Proteobacteria*, *Chloroflexi*, and *Fusobacteriota* decreased, while the abundance of *Actinobacteriota* and *Firmicutes* increased in the RTHtc group (Figure 4B). At the genus level, compared with the CONtc group, the relative abundance of *Methyloversatilis* and *Fusobacterium* increased, and the relative abundance of *Muribacter* and *Corynebacterium* decreased in the GUtc group. Compared with the GUtc group, the relative abundance of *Muribacter, Corynebacterium, Endozoicomonas, Porphyromonas,* and *Mycoplasma* increased. In contrast, the relative abundance of *Rodentibacter*, *Rothia*, *Streptococcus*, *Methyloversatilis*, and *Globicatella* decreased in the RTHtc group (Figure 4C). The analysis of variance (ANOVA) based on taxonomic composition and relative abundance showed that at the genus level, *Fusobacterium* and *Methyloversatilis* were significantly enriched in the GUtc group compared to the CONtc group. Conversely, *Muribacter*, *Endozoicomonas*, *Corynebacterium*, *Mycoplasma, unclassified_Spirochaetaceae*, and *unclassified_Alphaproteobacteria* were more abundant in the RTHtc groups than in the GUtc group (Figure 4D).

#### 3.3.2 Analysis of diversity indices in oral (tongue coating) microbiota

**Figure 5:**
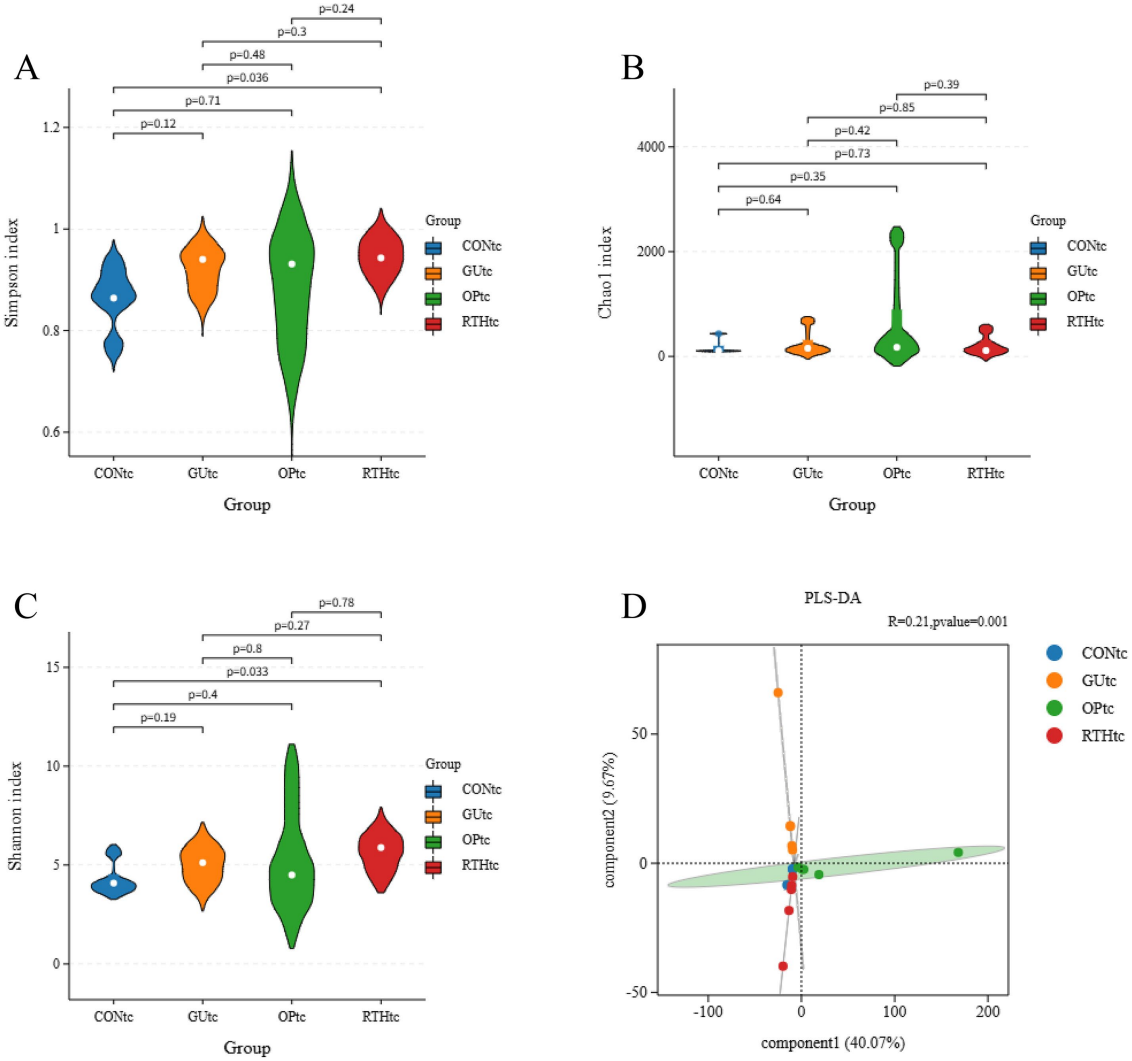
Alpha-diversity indices and partial least squares discriminant analysis of oral (tongue coating) microbiota. **(A)** Simpson index; **(B)** Chao1 index; **(C)** Shannon index; **(D)** PLS-DA.

To compare species diversity within microbial communities, *α*-diversity indices were employed to evaluate microbial richness and diversity. The Chao1 index, a standard metric for species richness, was used to estimate the number of OTUs. Simpson and Shannon–Wiener indices were applied to assess microbial diversity. Compared to the CONtc group, the GUtc, OPtc, and RTHtc groups exhibited progressively increased Shannon and Simpson indices, while no significant differences in the Chao1 index were observed. This suggests that modeling and pharmacological interventions induced an increase in OTU content within the tongue coating microbiota (Figures 5A–C). Partial least squares discriminant analysis (PLS-DA) revealed that the oral microbiota composition of the GUtc group rats deviated from that of the CONtc group. In contrast, the OPtc and RTHtc groups approached the distribution of the CONtc group. Components 1 and 2 explained 40.07 and 9.67% of the variance, respectively, revealing distinct differences (Figure 5D).

#### 3.3.3 Analysis of differential microbiota in oral (tongue coating)

**Figure 6:**
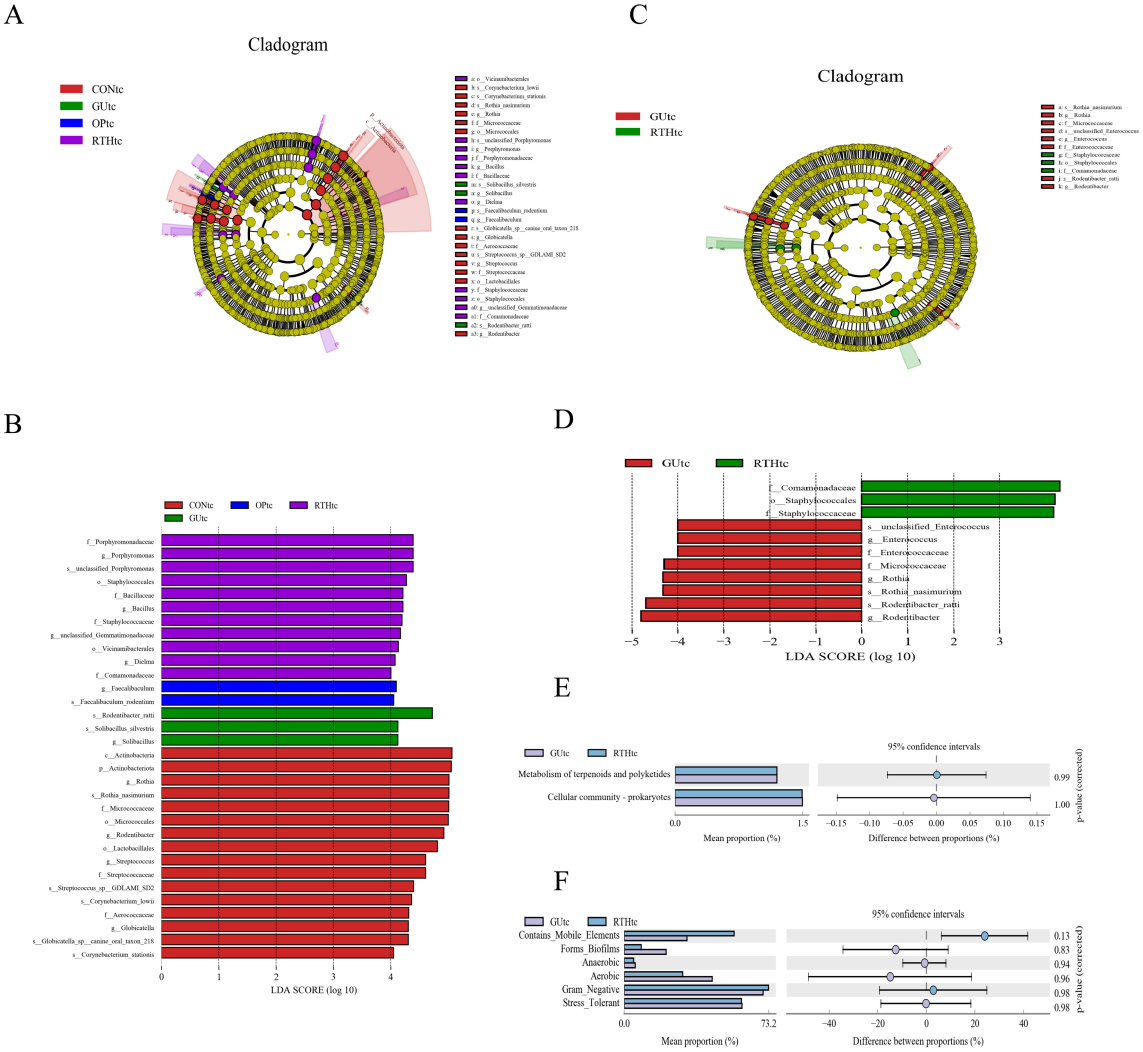
Oral (tongue coating) microbiota differential analysis and association analysis with gastric ulcer inflammatory factors. **(A)** LEfSe linear discriminant analysis (LDA) distribution histogram for all groups; **(B)** LEfSe cladogram illustrating taxonomic differences among all groups; **(C)** LDA distribution histogram comparing GUtc and RTHtc groups; **(D)** LEfSe cladogram comparing GUtc and RTHtc group differences; **(E)** Functional composition bar plot comparing GUtc and RTHtc groups; **(F)** Microbial community phenotypic analysis between the GUtc and RTHtc groups.

The LEfSe method integrates non-parametric Kruskal–Wallis and Wilcoxon rank–sum tests with LDA effect size to identify taxa with significant abundance differences across groups. This approach quantifies the effect of each microbial feature on group separation using LDA. In this study, LEfSe analysis was performed on tongue coating samples from all experimental groups, with a threshold LDA score >4 indicating statistically significant differences. When all groups were compared, dominant taxa included *Actinobacteria*, *Rothia*, and *Micrococcaceae* in the CONtc group; Enriched taxa included *Rodentibacter ratti*, *Solibacillus silvestris*, and *Solibacillus* in the GUtc group; Key discriminative taxa included *Faecalibaculum* and *Faecalibaculum rodentium* in the OPtc group; Predominant taxa included *Porphyromonadaceae*, *Porphyromonas*, and *Staphylococcales* in the RTHtc group (Figures 6A,B). When comparing the GUtc group with the RTHtc group, we found that differential taxa included in the GUtc group were *Rodentibacter*, *R. ratti*, and *Rothia*, while the main microbial communities in the RTHtc group were *Comamonadaceae*, *Staphylococcales*, and *Staphylococcaceae* (Figures 6C,D).

#### 3.3.4 KEGG metabolic pathway analysis of oral (tongue coating) microbiota

Functional prediction of the Kyoto Encyclopedia of Genes and Genomes (KEGG) metabolic pathways revealed compositional differences in the tongue coating microbiota of the GUtc group and RTHtc group. The predicted KEGG Level 2 pathways primarily involved metabolism of terpenoids and polyketides, and cellular community-prokaryotes. Functional disparity analysis between groups demonstrated enrichment in carbohydrate metabolism, membrane transport, and amino acid metabolism (Figure 6E). Utilizing BugBase for the classification of microbial communities into high-level phenotypes, it was observed that Gram-negative bacteria and mobile elements were more common in the RTHtc group than in the GUtc group (Figure 6F).

#### 3.3.5 Association between oral (tongue coating) microbiota and gastric ulcer clinical parameters

**Figure 7:**
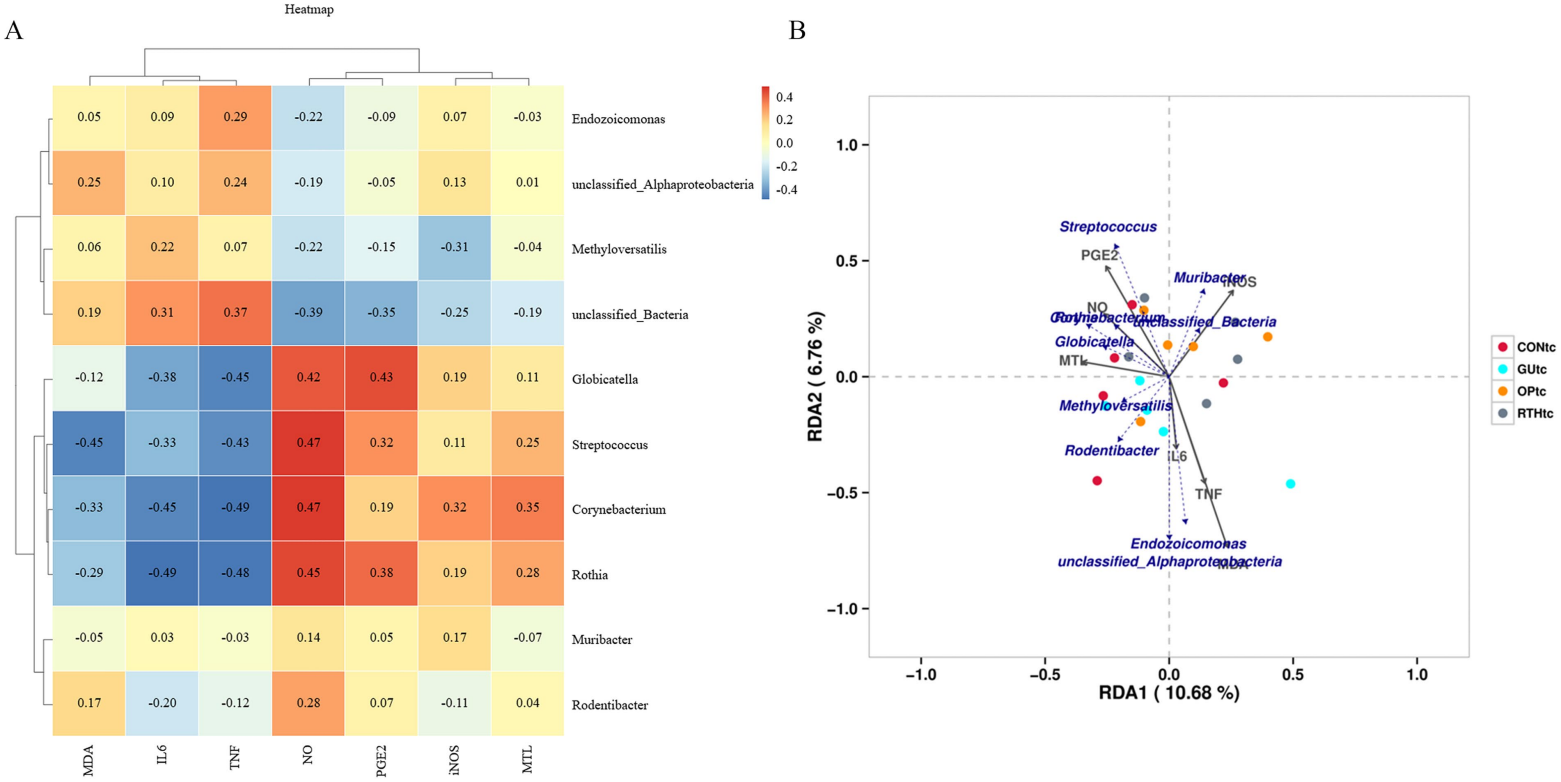
Association between oral microbiota and gastric ulcer. **(A)** Correlation heatmap between inflammatory factors and oral microbiota; **(B)** Redundancy analysis (RDA) plot depicting associations of inflammatory factors with oral microbiota.

To investigate the correlation between oral microbial community structure and gastric ulcer progression, a correlation heatmap analysis was performed between the oral microbiota and seven ulcer-related inflammatory factors (Figure 7A). The analysis revealed distinct microbial communities exerting significant effects on specific inflammatory markers. *Rothia*, *Corynebacterium*, and *Streptococcus* exhibited significant positive correlations with NO levels (*p <* 0.05), while *Globicatella*, *Corynebacterium*, and *Rothia* showed negative correlations with TNF-*α* (*p <* 0.05). Notably, *Corynebacterium* and *Rothia* were inversely associated with IL-6 (*p <* 0.05), and *Streptococcus* demonstrated a negative correlation with MDA (*p <* 0.05). Further redundancy analysis (RDA) was employed to quantify the relationship between inflammatory factors and oral bacterial community composition, with inflammatory factor expression values explaining 10.68% (Axis 1) and 6.76% (Axis 2) of the total microbial variation (Figure 7B). The RDA results corroborated the heatmap findings, confirming the critical role of *Rothia*, *Corynebacterium*, and *Streptococcus* in modulating inflammatory responses. These collective findings underscore the pivotal influence of oral microbial community structure on gastric ulcer-associated inflammatory processes. The results indicated that the bacteria enriched by RTHtc treatment may not merely be passive biomarkers; they actively contribute to the therapeutic outcome by modulating host immunity and ameliorating oxidative damage.

### 3.4 Analysis of gut microbiota results

#### 3.4.1 Identification and taxonomic distribution of gut microbiota

**Figure 8:**
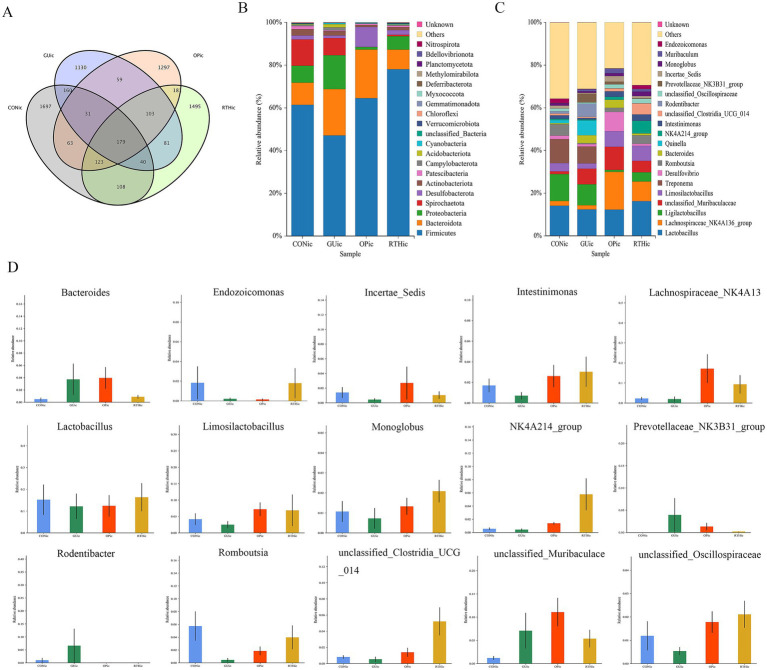
Analysis of gut microbiota composition and taxonomic distribution. **(A)** OTU clustering intersection Venn diagram; **(B)** Phylum-level relative abundance bar plot; **(C)** Genus-level relative abundance bar plot; **(D)** ANOVA of genus-level abundance.

To investigate the effects of RT on the gut microbiota structure in spleen–stomach damp–heat syndrome gastric ulcer rats, intestinal contents from the CON intestinal contents (CONic), GU intestinal contents (GUic), and RTH intestinal contents (RTHic) groups were analyzed. Bacterial 16S rDNA V3–V4 regions were amplified based on the prokaryotic small subunit ribosomal RNA (16S rRNA) gene sequences. OTUs were clustered at 97% similarity, yielding 6,747 OTUs across all 20 samples. A Venn diagram was employed to visualize shared and unique microbial taxa among groups, identifying core microbiota across experimental conditions. The GUic, CONic, OP intestinal contents (OPic), and RTHic groups contained 1,783, 2,401, 2,036, and 2,310 OTUs, respectively. Intersection analysis revealed 410 shared OTUs between GUic and CONic groups, 403 shared OTUs between GUic and RTHic groups, and 372 shared OTUs between GUic and OPic groups (Figure 8A).

At the phylum level, compared to the CONic group, the GUic group exhibited increased relative abundance of *Bacteroidota*, *Proteobacteria*, and *Acidobacteriota*, while showing decreased relative abundance of *Firmicutes* and *Spirochaetota*. In contrast, the RTHic group demonstrated elevated relative abundance of *Firmicutes*, *Desulfobacterota*, and *Patescibacteria*, alongside reduced relative abundance of *Bacteroidota*, *Proteobacteria*, and *Spirochaetota* relative to the GUic group (Figure 8B). At the genus level, the GUic group displayed higher relative abundance of *unclassified-Muribaculaceae*, *Bacteroides*, and *Quinella*, *Rodentibacter*, *Prevotellaceae_NK3B31_group*, and *Muribaculum* compared to the CONic group, whereas *Lactobacillus*, *Limosilactobacillus*, *Romboutsia*, *Lachnospiraceae-NK4A136-group* and *Ligilactobacillus* were significantly reduced. Conversely, the RTHic group demonstrated a higher relative abundance of *Lactobacillus*, *Romboutsia, the Lachnospiraceae-NK4A136-group*, and *Limosilactobacillus*, yet a lower relative abundance of *Ligilactobacillus*, *unclassified-Muribaculaceae*, and *Desulfovibrio* compared to the GU group (Figure 8C).

The ANOVA was performed to evaluate species composition and relative abundance of gut microbiota. At the genus level, the relative abundances of *unclassified_Muribaculaceae*, *Rodentibacter*, *Prevotellaceae_NK3B31_group*, and *Bacteroides* were elevated in the GUic group as compared to the CONic group, whereas they were reduced in the RTHic group in comparison to the GUic group (Figure 8D). In contrast, the relative abundances of *Monoglobus*, *unclassified_Oscillospiraceae, Incertae_Sedis*, *Intestinimonas*, *Limosilactobacillus*, *Lactobacillus*, *Endozoicomonas*, *Romboutsia*, *NK4A214_group*, *unclassified_Clostridia_UCG_014*, and *Lachnospiraceae_NK4A136_group* were enriched in the RTHic groups but depleted in the GUic group (Figure 8D). The results indicated that the microbiota in the GUic group exhibited a typical pattern of dysbiosis, characterized by a decrease in beneficial bacteria and an increase in potentially harmful bacteria. RTHic treatment not only corrected the microbiota dysbiosis caused by gastric ulcers but also shaped a beneficial microbiota structure, characterized by an enrichment of short-chain fatty acid-producing bacteria and probiotics.

#### 3.4.2 Analysis of gut microbiota diversity indices

**Figure 9:**
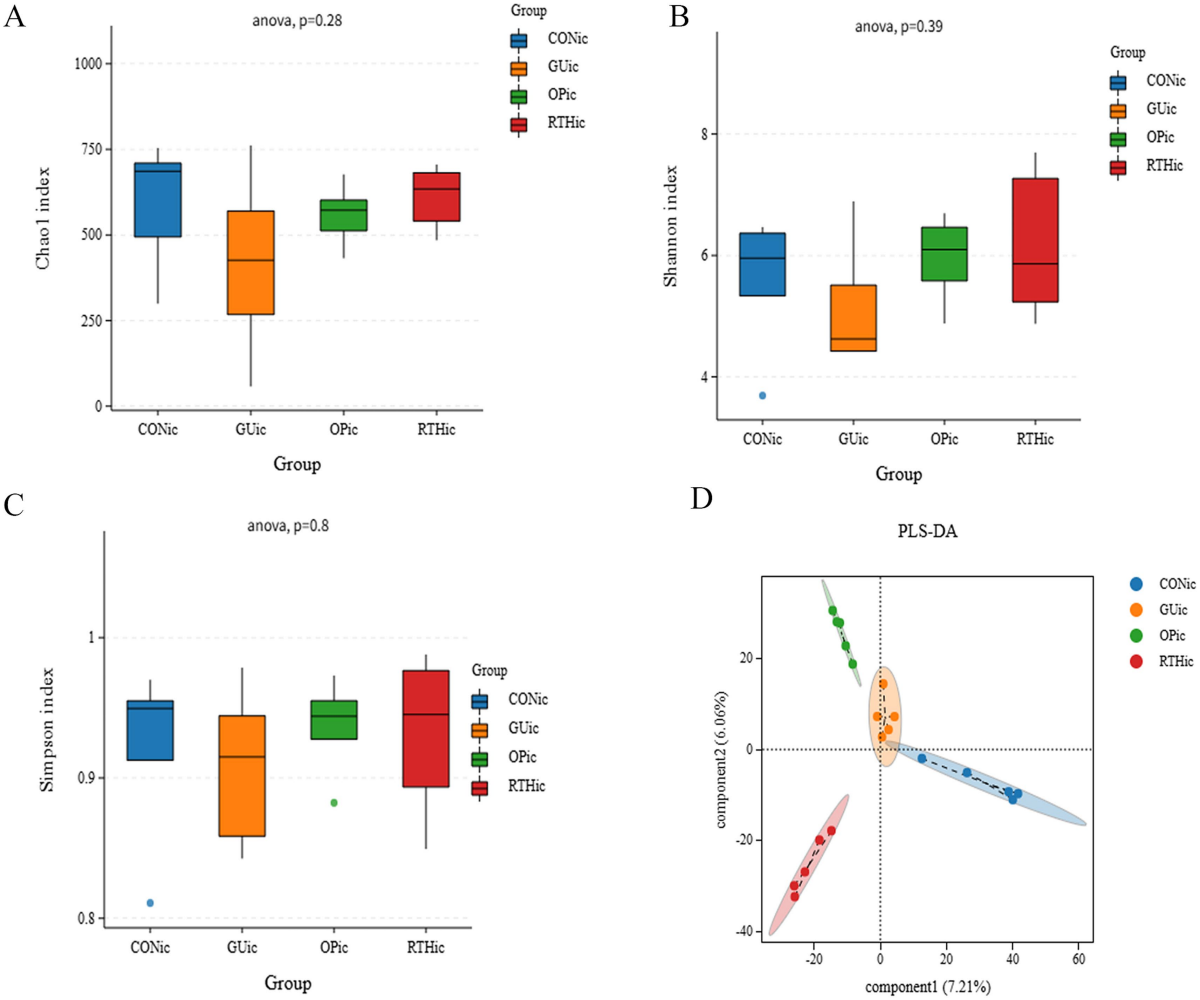
Analysis of gut microbiota alpha diversity indices and partial least squares discriminant analysis. **(A)** Chao1 index; **(B)** Shannon index; **(C)** Simpson index; **(D)** PLS-DA.

Compared with the CONic group, the GUic group exhibited significant reductions in Simpson, Chao 1, and Shannon indices, indicating diminished microbial diversity and richness. In contrast, both the OPic and RTHic groups showed marked recovery of these indices, with values approaching those of the CONic group (Figures 9A–C). PLS-DA revealed an altered gut microbiota composition in the GUic group, with its distribution deviating from that of the CONic group. At the same time, the microbial distributions of the OPic and RTHic groups were also distant from the CONic group. Components 1 and 2 explained 7.21 and 6.06% of the variance, respectively, demonstrating distinct intergroup differences (Figure 9D).

#### 3.4.3 Analysis of differential gut microbiota in intestinal contents

**Figure 10:**
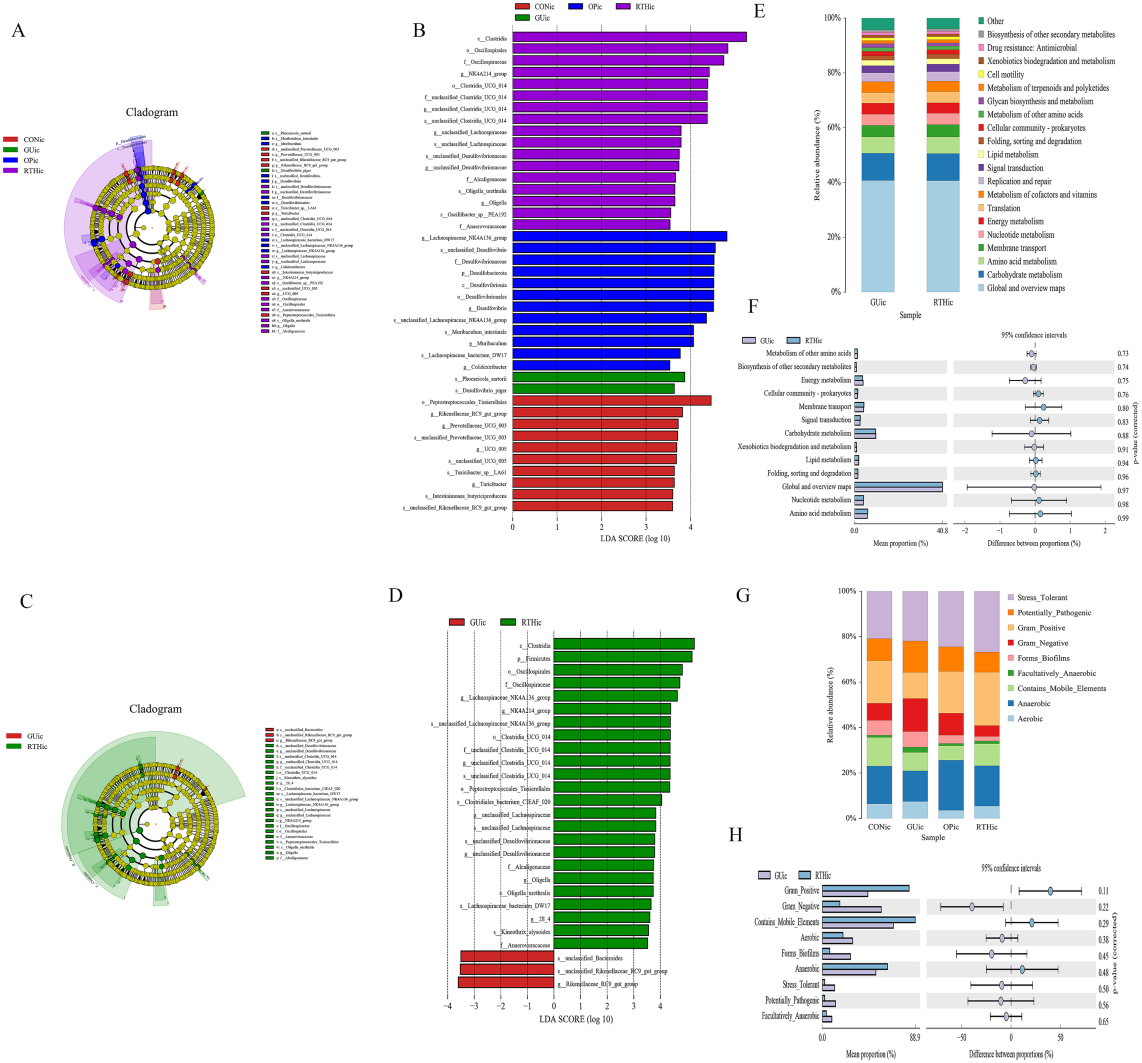
Gut microbiota differential analysis. **(A)** LEfSe cladogram illustrating taxonomic distinctions among all groups; **(B)** LEfSe linear discriminant analysis (LDA) distribution histogram across all groups; **(C)** LEfSe cladogram comparing GUic and RTHic group differences; **(D)** LDA distribution histogram comparing GUic and RTHic groups; **(E)** Functional composition bar plot comparing the GUic and RTHic groups; **(F)** Functional divergence analysis between the GUic and RTHic groups; **(G)** Microbial community phenotypic bar plot; **(H)** Microbial community phenotypic analysis between the GUic and RTHic groups.

LEfSe analysis was performed on intestinal content samples across groups, with a linear discriminant analysis (LDA) score threshold of >4 indicating statistically significant intergroup differences. Comparative analysis revealed distinct microbial biomarkers for each group. Dominant taxa included *Tissierellales*, *Rikenellaceae_RC9_gut_group*, and *Prevotellaceae-UCG-003* in the CON group; Key biomarkers were *Phocacicola-sartorii* and *Desulfovibrio-piger* in the GUic group; Predominant microbiota comprised *Lachnospiraceae-NK4A136-group*, *Desulfovibrionaceae*, and *Desulfovibrio* in the OPic group; Enriched taxa included *Clostridia*, *Oscillatoriales*, and *Oscillospiraceae* in the RTHic group (Figures 10A,B). When comparing GUic and RTHic groups, *unclassified-Bacteroides* and *Rikenellaceae-RC9-gut-group* were identified as biomarkers in the GUic group, while RTHic group biomarkers remained consistent with the aforementioned taxa (Figures 10C,D).

#### 3.4.4 KEGG metabolic pathway analysis of gut microbiota

Functional composition analysis of KEGG metabolic pathways between the GUic group and RTHic groups revealed that the predicted secondary KEGG metabolic pathways were found to be analogous to those of the tongue coating microbiota (Figures 10E,F), indicating a potential microbial crosstalk between the gut and oral ecosystems in GUic pathogenesis. Functional divergence analysis further demonstrated enrichment in amino acid metabolism, Nucleotide metabolism, lipid metabolism, carbohydrate metabolism, and energy metabolism, among others. Notably, the dysregulation of gut microbiota in GUic rats exhibited a strong association with perturbations in *nucleotide metabolism*, suggesting its critical role in GU-related microbial dysbiosis. Utilizing BugBase for the classification of microbial communities into high-level phenotypes, it was observed that Gram-positive, mobile element-containing, and anaerobic phenotypes were more prevalent in the RTHic group than in the GUic group (Figures 10G,H).

#### 3.4.5 Association between gut microbiota and gastric ulcer clinical parameters

**Figure 11:**
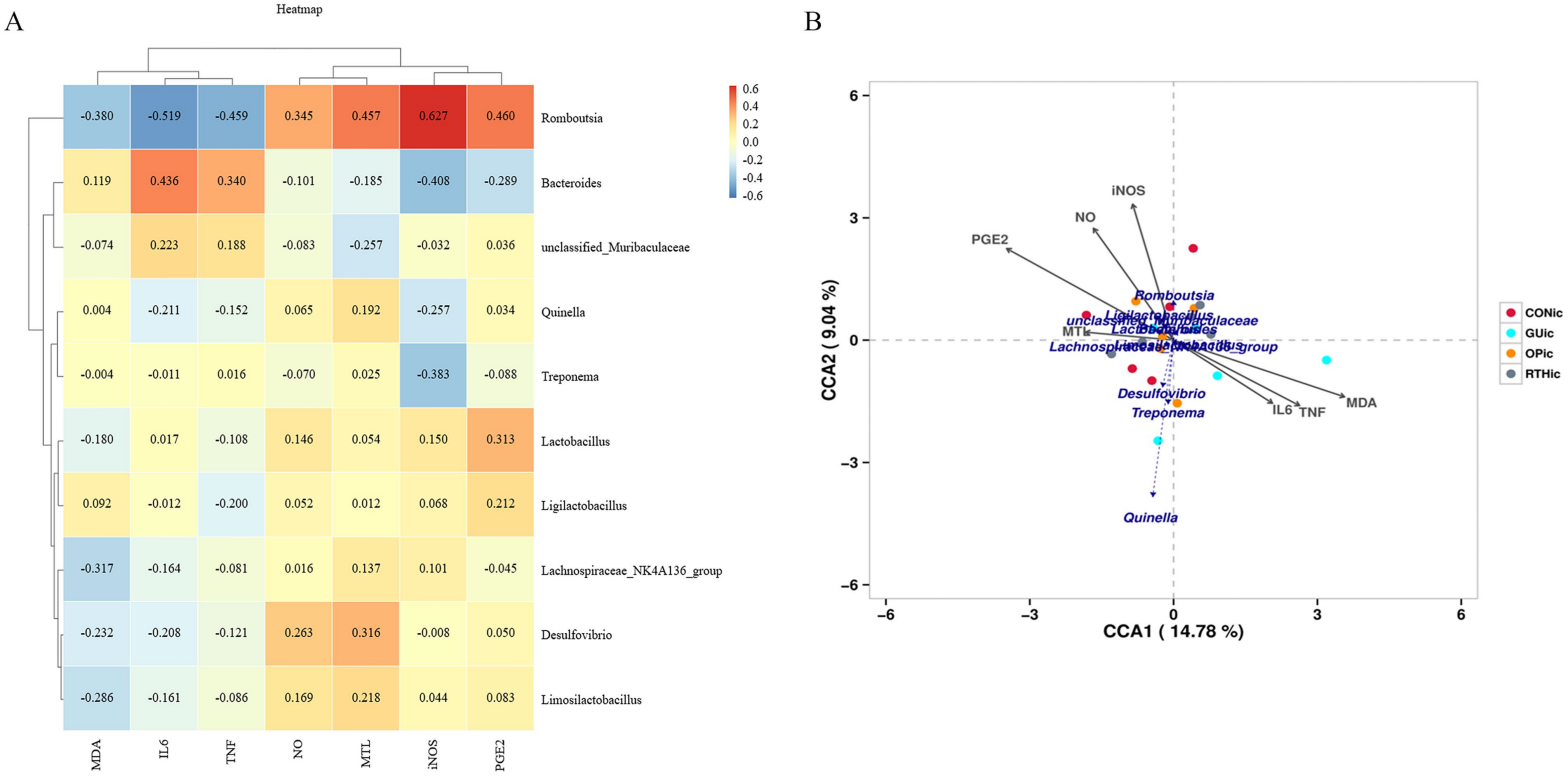
Association between gut microbiota and gastric ulcer. **(A)** Correlation heatmap between inflammatory factors and gut microbiota; **(B)** canonical correspondence analysis (CCA) plot depicting associations of inflammatory factors with gut microbiota.

To investigate the correlation between gut microbial community structure and gastric ulcer progression, a correlation heatmap analysis was performed (Figure 11A). The analysis identified significant associations between specific gut microbiota and inflammatory mediators. *Romboutsia* exhibited positive correlations with iNOS, PGE_2_, and MTL (*p <* 0.05), while demonstrating negative correlations with IL-6 and TNF-*α* (*p <* 0.05). Further canonical correspondence analysis (CCA) revealed that inflammatory factor expression values accounted for 14.78% (Axis 1) and 9.04% (Axis 2) of the total microbial community variation (Figure 11B). The CCA ordination demonstrated that *Romboutsia*, *Ligilactobacillus*, *Limosilactobacillus*, and *Lactobacillus* were positively associated with iNOS, PGE_2_, NO, and MTL, but inversely correlated with IL-6, MDA, and TNF-α. Conversely, *Treponema*, *Quinella*, and *Desulfovibrio* showed opposite correlation patterns with these inflammatory markers. These findings collectively highlight the critical involvement of gut microbial community structure in modulating inflammatory responses during gastric ulcer pathogenesis. Beneficial bacteria, such as *Lactobacillus* and *Romboutsia*, are positively correlated with gastric mucosal protective factors (PGE_2_ and NO) and negatively correlated with pro-inflammatory factors (IL-6 and TNF-α). They may serve as effective strategies for treating gastric ulcers through the use of probiotics and prebiotics. In contrast, harmful bacteria like *Treponema* and *Desulfovibrio* exhibit the opposite pattern; their increase or decrease is related to the inflammatory gastric environment.

### 3.5 Analysis of dominant taxa in oral (tongue coating) and gut microbiota

**Figure 12:**
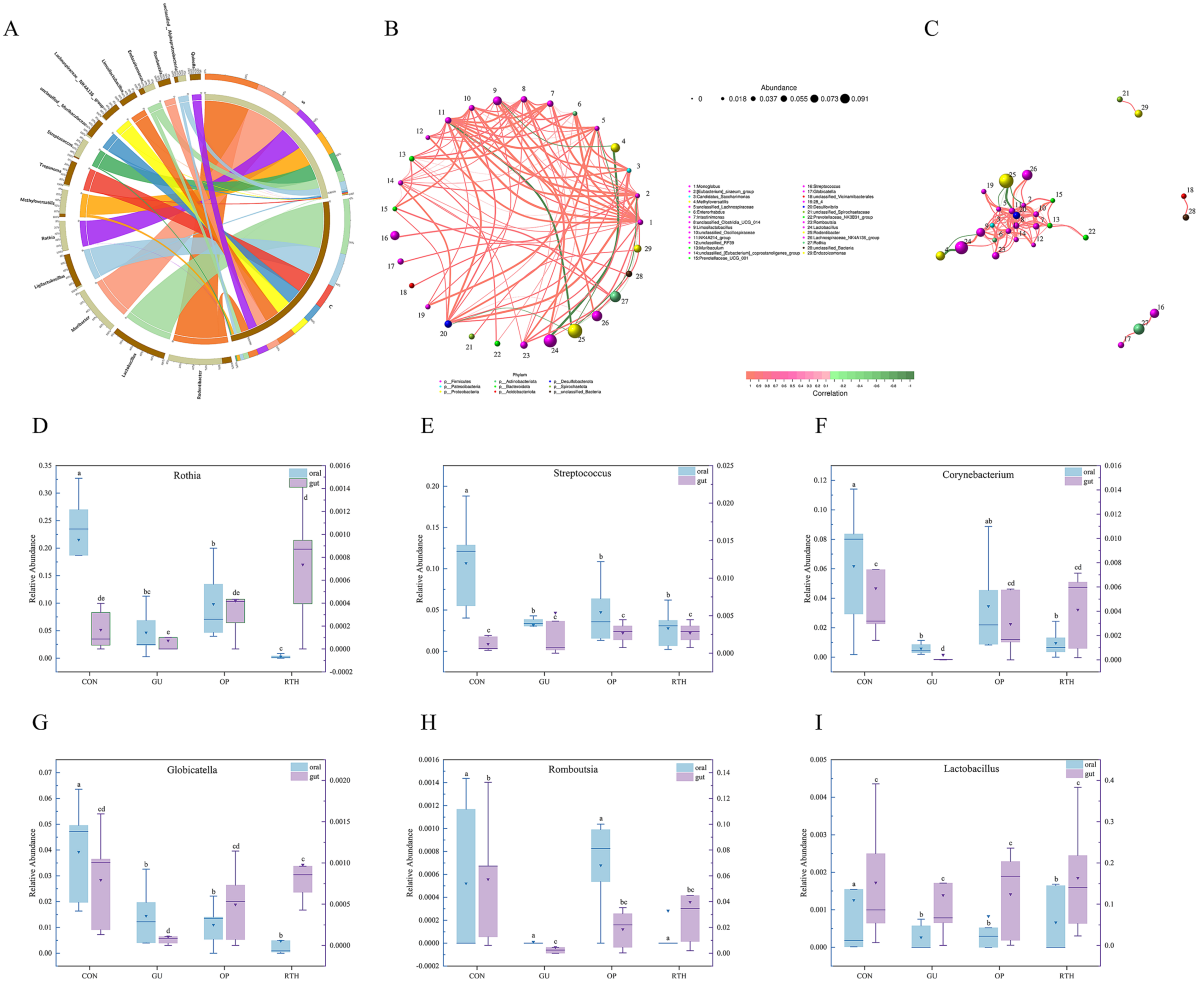
Correlation analysis between oral (tongue coating) and gut microbiota. **(A)** Circos diagram illustrating species composition of oral–gut microbiota; **(B)** network diagram of oral–gut microbial layout correlation; **(C)** network diagram of oral–gut microbial module correlation; **(D)** *Rothia*; **(E)** *Streptococcus*; **(F)** *Corynebacterium*; **(G)** *Globicatella*; **(H)** *Romboutsia*; **(I)** *Lactobacillus*.

The Circos diagram depicts the correlation between tongue coating, intestinal contents, and microbial species, illustrating the proportional composition of dominant species within samples and their distribution across various sample types. In the diagram, colored ribbons indicate the presence of specific species in a sample, with the width of the ribbons corresponding to the relative abundance of the species, and wider ribbons denote a higher abundance. As depicted in Figure 12A, *Rothia*, *Rodentibacter*, *Methyloversatilis*, *Streptococcus*, and *Endozoicomonas* were predominantly identified in tongue coating samples, representing the dominant genera of the tongue microbiota. Notably, *Muribacter* was exclusively detected in tongue coating samples, serving as a distinctive genus of this niche. In contrast, *Ligilactobacillus*, *Treponema*, *Lachnospiraceae-NK4A136-group, Lactobacillus*, *Limosilactobacillus*, *Romboutsia*, and *Quinella* were primarily enriched in intestinal samples, forming the dominant intestinal genera.

### 3.6 Oral (tongue coating)–gut microbial correlation analysis

The correlation network diagram visually illustrates the relationship between tongue coating and gut microbiota at the genus level. Spearman correlation analysis was used to evaluate interspecies relationships based on species abundance and dynamic variations across samples, followed by statistical validation. Key topological properties of the network, including node degree distribution, network diameter, average shortest path, and centrality metrics (Degree, Closeness Centrality, Betweenness Centrality) were calculated to characterize intra- and intergroup microbial interactions. *Monoglobus* exhibited significant positive correlations (Figure 12B; *p <* 0.05) with specific genera such as *Lachnospiraceae-NK4A214-group*, *unclassified-Clostridia-UCG-014*, *Intestinimonas*, *Romboutsia*, *unclassified-Oscillospiraceae*, *Muribaculum*, and *Endozoicomonas*. A strong positive correlation was also observed between *Limosilactobacillus* and *Lactobacillus* (Figure 12B; *p <* 0.05). Conversely, *Methyloversatilis* displayed significant negative correlations with *Limosilactobacillus* and *Lactobacillus* (Figure 12B; *p <* 0.05). Streptococcus showed positive associations with *Rothia* and *Globicatella* (Figure 12B; *p <* 0.05), whereas *Rodentibacter* was negatively correlated with *Endozoicomonas*, *Monoglobus*, and *Lachnospiraceae-NK4A214-group* (Figure 12B; *p <* 0.05). The oral bacteria, including *Rothia* and *Globicatella*, demonstrated strong synergistic interactions with *Streptococcus* (Figure 12C). Meanwhile, the gut bacterium *Romboutsia* exhibited positive correlations with multiple taxa, such as *Limosilactobacillus*, *Enterorhabdus*, and *Candidatus-Saccharimonas*. These findings highlight niche-specific microbial co-regulation patterns and potential functional synergies or antagonisms within the oral–gut axis.

### 3.7 Expression patterns of key microorganisms in oral–gut axis

At the genus level, *R. roxburghii* root extract modulated the composition of oral–gut microbiota in rats, with taxonomic restructuring showing intergroup variations (Figures 12D–I). Following gastric ulcer induction, the oral cavity exhibited sustained reductions in relative abundances of *Rothia*, *Streptococcus*, *Corynebacterium*, *Globicatella*, *Romboutsia*, and *Lactobacillus*. Notably, therapeutic administration of *R. roxburghii* root extract significantly enhanced intestinal abundances of beneficial taxa, including *Rothia*, *Streptococcus*, *Corynebacterium*, *Globicatella*, and *Romboutsia*. Compared to the CON group, GU rats showed marked decreases in oral relative abundances of *Rothia*, *Streptococcus*, *Corynebacterium*, *Globicatella*, and *Lactobacillus* (*p <* 0.05). Conversely, in the gut microenvironment, *R. roxburghii* root-treated (RTH) group demonstrated significant recovery of *Rothia* and *Globicatella* abundances compared to GU controls (*p <* 0.05). These differential expression patterns across anatomical niches highlight the bidirectional regulatory capacity of *R. roxburghii* root on microbial homeostasis during ulcer pathogenesis.

## 4 Discussion

Gastric ulcer (GU), a common digestive disorder, is clinically characterized as spleen–stomach damp–heat syndrome, a subtype of GU. As described, internal and external pathogenic factors interact synergistically, resulting in damp–heat disorders. Multiple etiological factors induce inflammatory responses and localized necrosis of the mucosal muscular layer through coordinated actions, ultimately leading to the formation of GU. Studies indicate that GU primarily localizes in the gastrointestinal tract, with its pathogenesis linked to functional dysregulation of the liver, spleen, stomach, and intestines (Zhou et al., 2024). During the active phase of GU, the core pathogenesis involves “heat-toxin” accumulation in the spleen–stomach complex, characterized by damp–heat retention, obstructed qi dynamics, and impaired middle-jiao function. Prolonged damp–heat stagnation leads to tissue necrosis and ulceration. In recent years, Traditional Chinese medicine (TCM) has gained prominence in treating digestive disorders due to its principle of “addressing acute symptoms first while targeting root causes gradually,” offering advantages over antibiotic-associated adverse effects (Xia et al., 2021; Yang et al., 2022). This study represents the first comprehensive identification of primary bioactive constituents in *R. roxburghii* root (RT) using LC–MS/MS technology. The quantitative analysis confirmed that the RT material met predefined quality standards, with four marker compounds including ellagic acid, gallic acid, ferulic acid, and rutin as validated chemical benchmarks for subsequent experimental investigations. Subsequently, guided by TCM theory, this study established a rat model of the spleen–stomach damp–heat syndrome GU through a combined high-fat/high-sugar diet and damp–heat environmental exposure, effectively replicating clinical disease manifestations (Sedghi et al., 2021). Following successful model establishment, gastric tissue histopathology revealed distinct pathological features of gastric ulcers, with a significant increase in the gastric mucosal injury index. The GU group exhibited elevated levels of inflammatory factors IL-6, TNF-*α*, MDA, and reduced levels of MTL and PGE_2_ compared to the CON group, all demonstrating statistically significant differences (*p <* 0.05), thereby confirming successful modeling. The levels of pro-inflammatory cytokines TNF-α and IL-6, along with the oxidative stress product MDA, increased, exacerbating mucosal inflammation and oxidative damage. Simultaneously, the levels of the key mucosal defense factor PGE_2_ and MTL, which regulates gastrointestinal motility, significantly decreased. This weakening of the mucosal self-protection and clearance ability jointly promotes the formation of gastric ulcers. After treatment with the RT, the levels of TNF-α, IL-6, and MDA rapidly decreased, and the level of PGE_2_ was restored. This favorable microenvironment promoted repair at the histological level.

The human microbial ecosystem is distributed across nearly all body cavities and open surfaces, with digestive disease-associated microbiota encompassing oral, gastric, and intestinal microbial communities (Hou et al., 2022; Chen, 2016). The dynamic alterations along the oral–gastric–intestinal microbial axis hold significant research value for understanding gastrointestinal pathologies. As a cornerstone of TCM syndrome differentiation, tongue diagnosis bridges the study of oral (tongue coating) microbiota and gut microbiota, providing theoretical support for standardizing TCM disease classification and syndrome differentiation. This integration advances mechanistic insights into disease progression while guiding early diagnosis and therapeutic strategies. Notably, oral microbial shifts may directly influence tongue-coating morphology and microbiota composition, establishing a bidirectional interaction. Thus, investigating GU through either oral or tongue coating microbiota offers complementary perspectives (Du et al., 2025). In this study, the dominant phylum in GUtc group tongue coating samples included *Proteobacteria*, *Bacteroidota*, *Acidobacteriota*, *Chloroflexi*, and *Fusobacteriota*. At the genus level, the GUtc group exhibited increased relative abundances of *Methyloversatilis* and *Fusobacterium* but decreased *Muribacter* and *Corynebacterium*. Compared to the GUtc group, the RTHtc group exhibited elevated levels of *Muribacter*, *Corynebacterium*, *Endozoicomonas*, *Porphyromonas*, and *Mycoplasma,* while concurrently showing reduced levels of *Rodentibacter*, *Rothia*, *Streptococcus*, *Methyloversatilis*, and *Globicatella*. Notably, *Methyloversatilis* and *Fusobacterium*, which are anaerobic Gram-negative pathogens, are frequently detected in oral and dental plaques as well as the colon. These pathogens are associated with various conditions, including periodontitis, acute necrotizing gingivitis, oral cancer, ulcerative colitis, and colorectal cancer (Zhu et al., 2024). *Corynebacterium* refers to Gram-positive bacilli lacking spores, with most strains being non-motile. As a beneficial oral bacterium, it contributes to maintaining oral microbial equilibrium (Yang et al., 2013). *Rothia*, a symbiotic genus constituting 2–31% of the total microbiota in healthy intestines, produces short-chain fatty acids (SCFAs), particularly butyrate, which exhibit demonstrated anti-inflammatory properties. This genus may play a critical role in modulating inflammatory processes, especially within the gastrointestinal tract (Mehdi, 2021). *Streptococcus*, a genus of Gram-positive pyogenic cocci, predominantly colonizes the nasopharynx and gastrointestinal tract as commensal flora. While most strains are non-pathogenic, certain species can induce suppurative infections and hypersensitivity disorders (Belgacem et al., 2025). The observed microbial shifts reflect a reduction in beneficial bacteria and proliferation of pathogenic taxa within the oral microbiota of GU rats, concurrently indicating a strong interconnection between oral dysbiosis and gut microbiota perturbations in the GU group. Notably, dysbiosis was alleviated following administration of *R. roxburghii* root extract. We hypothesize that this oral microbiota restoration may correlate with functional metabolic pathways, including carbohydrate metabolism and amino acid metabolism.

Emerging evidence highlights the close relationship between gut microbiota and GU, where modulating microbial diversity and restoring ecological balance may ameliorate ulcer progression (Nath et al., 2022). In a healthy gut microenvironment, beneficial and pathogenic bacteria coexist dynamically to maintain microbial homeostasis. However, disruption of this equilibrium allows conditional pathogens to proliferate and mediate inflammatory cascades (Fan and Pedersen, 2021). Dominant phyla in the gut microbiota include Firmicutes, Bacteroidota, Proteobacteria, and Spirochaetota. At the genus level, the GUic group exhibited increased relative abundances of *unclassified-Muribaculaceae*, *Bacteroides*, and *Quinella* alongside decreased *Lactobacillus*, *Romboutsia, Lachnospiraceae-NK4A136-group*, and *Ligilactobacillus* compared to the CONic group. Conversely, the RTHic intervention restored *Lactobacillus*, *Romboutsia, Lachnospiraceae-NK4A136-group*, and *Limosilactobacillus* while reducing *Ligilactobacillus*, *unclassified-Muribaculaceae*, and *Desulfovibrio*. These shifts suggest that GU-induced gastric mucosal atrophy or erosion perturbs intestinal homeostasis, altering the abundance of dominant taxa and microbial clustering structures (Gomaa, 2020). Correlation analysis between oral–gut microbiota and seven gastric ulcer-related inflammatory factors revealed that significant alterations in inflammatory factor levels following ulcer onset directly induced marked reductions in the relative abundances of oral bacteria *Rothia*, *Corynebacterium*, and *Streptococcus*, as well as gut microbiota *Romboutsia* (*p <* 0.05). Notably, restoration of microbial homeostasis through *R. roxburghii* root treatment conversely modulated inflammatory factor expression. These findings were corroborated by both RDA and CCA. In the study exploring the correspondence between oral and intestinal flora, it was found that the oral bacterium *Methyloversatilis* was significantly negatively correlated with the abundance of the characteristic intestinal genera *Limosilactobacillus* and *Lactobacillus* (*p <* 0.05). In addition, there was a close interaction between oral bacteria *Rothia*, *Globicatella*, and *Streptococcus*, and it was positively regulated; intestinal bacteria *Romboutsia* were positively correlated with a variety of bacteria, such as *Limosilactobacillus*, *Enterorhabdus*, *Candidatus-Saccharimonas*, etc., which were positively correlated. The expression of inflammatory mediators can directly reflect the degree of damage to the gastric mucosa, so when gastric ulceration occurs there will be a decrease in the levels of NO, iNOS, PGE_2_ and MTL, and an increase in the levels of TNF-*α*, IL-6 and MDA, and the significant changes in the levels of these inflammatory factors will directly lead to a significant decrease in the abundance of some oral and intestinal bacteria (e.g., *Rothia*, *Corynebacterium*, *Streptococcus*, *Globicatella*, *Lactobacillus*, *Romboutsia*). The increase in the abundance of these bacteria after treatment with *R. roxburghii* root may improve the clinical symptoms of gastric ulcers by directly modulating the levels of gastric ulcer-associated inflammatory factors. In conclusion, the oral microbiota downregulates gut microbial communities, while gut-derived ecological perturbations reciprocally reshape oral bacteriome composition. Bidirectional oral–gut microbial crosstalk critically modulates gastric ulcer pathogenesis and resolution.

## 5 Conclusion

This study successfully established a gastric ulcer model of spleen and stomach damp–heat syndrome. Following treatment with *R. roxburghii* Root (RT), the levels of MDA, IL-6, and TNF-α significantly decreased, while the levels of mucosal protective factors PGE_2_, iNOS, and MTL increased. This indicates that RT inhibits inflammatory responses and oxidative stress, enhances mucosal protection, and promotes ulcer repair. Analysis of tongue coating microbiota revealed that the abundances of *Muribacter* and *Corynebacterium*, along with other genera, increased after RT intervention, and the microbiota structure approached that of the control group. Moreover, *Corynebacterium* and other genera were significantly negatively correlated with TNF-α and IL-6, suggesting that RT may regulate the structure of tongue coating microbiota to control immunity and oxidative damage. Analysis of intestinal microbiota showed that after RT intervention, the Firmicutes phylum, *Lactobacillus* genus, *Romboutsia*, and other short-chain fatty acid-producing and probiotic bacteria were significantly enriched, and the microbiota structure was separated significantly. Correlation analysis revealed that *Romboutsia* and *Lactobacillus*, among others, were positively correlated with mucosal protective factors and negatively correlated with inflammatory factors, while harmful bacteria such as *Treponema* and *Desulfovibrio* showed the opposite correlation. The results indicated that RT can remodel the structure of tongue coating and intestinal microbiota, promote beneficial bacteria, inhibit harmful bacteria, and regulate microbial metabolic functions and host inflammatory responses, thereby improving gastric ulcers. These findings substantiated that gastric ulcer pathology, at the oral–gut microecological level, manifests objective biological underpinnings corresponding to TCM syndrome patterns.

## Data Availability

The data presented in the study are deposited in the NCBI repository, accession number PRJNA1337212.

## References

[ref1] Belgacem S. Chaâbane-Banaoues R. Mejri A. Ifa S. B. Mastouri M. Babba H. (2025). Parasitological and microbiological assessment of contact lens storage cases: a survey of asymptomatic lens student wearers from five medical specialties in Tunisia, North Africa. BMC Infect. Dis. 25:227. doi: 10.1186/s12879-024-10357-5, PMID: 39956912 PMC11831826

[ref2] Cao M. Zhang B. Yang Q. Liu Y. Cao S. J. Kang N. (2025). Mechanism of Rhizoma coptidis water extract in the treatment of the rats of gastric ulcer of spleen-stomach damp-heat type. J. Tianjin Univ. Trad. Chin. Med. 44, 21–30. doi: 10.11656/j.issn.1673-9043.2025.01.05

[ref3] Chen H. (2016). The composition of the oral microbiome and its relationship with human diseases. Shanghai: Shanghai Jiao Tong University.

[ref4] Chen J. Z. Meng Q. F. Chen J. H. Peng Y. Z. Liu X. J. (2001). The experiment study of chronic gastric ulcer treatment with root of rose Roxburghii tratt in rats. Guizhou Medi. J. 25, 584–585.

[ref5] Chen S. Tang Y. H. Gao Y. Nie K. X. Wang H. Z. Su H. . (2022). Antidepressant potential of quercetin and its glycoside derivatives: a comprehensive review and update. Front. Pharmacol. 13:865376. doi: 10.3389/fphar.2022.865376, PMID: 35462940 PMC9024056

[ref6] Du Q. J. Wu L. P. Zhang F. Dai P. X. Feng X. C. Zhang X. K. (2025). Difference analysis of oral flora in dogs with periodontitis and drug resistance of oral porphyromonas. Acta Veterinaria et Zootechnica Sinica. 56, 934–942. doi: 10.11843/j.issn.0366-6964.2025.02.041

[ref7] Fan Y. Pedersen O. (2021). Gut microbiota in human metabolic health and disease. Nat. Rev. Microbiol. 19, 55–71. doi: 10.1038/s41579-020-0433-9, PMID: 32887946

[ref8] Fu B. Chen J. Z. Fan S. Bao Q. C. Wan J. Hong J. X. (2022). Experimental study on the effect of Qingzhong Yuyang decoction on gastric ulcer of spleen and stomach damp-heat type. Medical Innov. China. 19, 28–34. doi: 10.3969/j.issn.1674-4985.2022.16.007

[ref9] Gomaa E. Z. (2020). Human gut microbiota/microbiome in health and diseases: a review. Antonie Van Leeuwenhoek 113, 2019–2040. doi: 10.1007/s10482-020-01474-7, PMID: 33136284

[ref10] Gong H. Y. Zhao N. Zhu C. L. Luo L. Liu S. (2024). Treatment of gastric ulcer, traditional Chinese medicine may be a better choice. J. Ethnopharmacol. 324:117793. doi: 10.1016/j.jep.2024.117793, PMID: 38278376

[ref11] Guizhou Provincial Drug Administration (2003). Quality standards for Chinese herbal medicines and ethnomedicinal herbs in Guizhou Province. Guiyang: Guizhou Science and Technology Press.

[ref12] Hossen J. M. Chou J. Y. Li S. M. Fu X. Q. Yin C. L. Guo H. . (2018). An ethanol extract of the rhizome of Atractylodes chinensis exerts anti-gastritis activities and inhibits Akt/NF-κB signaling. J. Ethnopharmacol. 228, 18–25. doi: 10.1016/j.jep.2018.09.015 30218812

[ref13] Hou K. Wu Z. X. Chen X. Y. Wang J. Q. Zhang D. Xia o C. . (2022). Microbiota in health and diseases. Signal Transduct. Target. Ther. 7:135. doi: 10.1038/s41392-022-00974-4, PMID: 35461318 PMC9034083

[ref14] Li L. L. (2018). Rosa Roxburghii Radix quality standard improvement and study on the pollution and distribution characteristics of heavy metals in soils and different parts of Rosa Roxburghii Tratt. Guizhou: Guizhou Normal University.

[ref15] Li L. Du Y. Y. Wang Y. He N. Wang B. Zhang T. (2022). Atractylone alleviates ethanol- induced gastric ulcer in rat with altered gut microbiota and metabolites. J. Inflamm. Res. 15, 4709–4723. doi: 10.2147/JIR.S372389, PMID: 35996682 PMC9392477

[ref16] Li X. F. Liu S. L. Zhang Y. M. Huang C. H. Luo D. D. (2025). Bletilla ochracea Schltr. protects against ethanol-induced acute gastric ulcers by alleviating oxidative stress and inflammation and modulating gut microbiota. Fitoterapia 181:106397. doi: 10.1016/j.fitote.2025.106397 39848595

[ref17] Liang Y. Li L. Q. Wang L. Zhou L. Yang X. S. (2022). Chemical constituents and their anti-inflammatory activities from rhizome of ethnic medicine Rosa roxburghii. Guihaia. 42, 1531–1541. doi: 10.11931/guihaia.gxzw202105005

[ref18] Liu Z. Y. Li Z. Y. Zhu G. B. Liu Y. Q. Peng Q. H. Wu Z. Z. (2023). Perturbations in gastrointestinal tract microbiota composition and function in individuals with yellow-greasy tongue coating. Digital Chin. Med. 6, 160–169. doi: 10.1016/j.dcmed.2023.07.006

[ref19] Luo H. J. Zhang Y. K. Yao Y. Zhang Q. L. Lin S. Q. Wang S. Z. . (2025). A novel galactoglucan from Ganoderma lucidum ameliorates ethanol-induced gastric ulcers by modulating FAK-MAPK signaling pathway. Carbohydr. Polym. 360:123594. doi: 10.1016/j.carbpol.2025.123594, PMID: 40399003

[ref20] Ma Y. C. (2020). Anti-escherichia coli and anti-inflammatory activity of ethyl acetate extract from the root of Rosa roxburghii tratt. Jiangsu: Nanjing Agricultural University.

[ref21] Mehdi B. F. (2021). Characterization of the Rothia spp. and their role in human clinical infections. Infect. Genet. Evol. 93:104877. doi: 10.1016/j.meegid.2021.104877 33905886

[ref22] Nath A. N. Retnakumar R. J. Francis A. Chhetri P. Thapa N. Chattopadhyay S. (2022). Peptic ulcer and gastric cancer: is it all in the complex host-microbiome interplay that is encoded in the genomes of “us” and “them”? Front. Microbiol. 13:835313. doi: 10.3389/fmicb.2022.835313, PMID: 35547123 PMC9083406

[ref23] Qu L. H. Liu C. L. Ke C. Zhan X. Li L. Q. Xu H. Y. . (2022). Atractylodes lancea rhizoma attenuates DSS-induced colitis by regulating intestinal flora and metabolites. Am. J. Chin. Med. 50, 525–552. doi: 10.1142/S0192415X22500203, PMID: 35114907

[ref24] Sedghi L. Dimassa V. Harrington A. Lynch S. V. Kapila Y. L. (2021). The oral microbiome: role of key organisms and complex networks in oral health and disease. Periodontol. 87, 107–131. doi: 10.1111/prd.12393, PMID: 34463991 PMC8457218

[ref25] Shen Y. Fan N. R. Ma S. X. Cheng X. Yang X. S. Wang G. (2025). Gut microbiota dysbiosis: pathogenesis, diseases, prevention, and therapy. MedComm 6:e70168. doi: 10.1002/mco2.70168, PMID: 40255918 PMC12006732

[ref26] Song X. L. Shi X. R. Zhang H. Li N. (2019). Professor Li Zhengsheng’s clinical experience in treating peptic ulcer using the spleen-stomach-liver dynamic syndrome differentiation method. Traditional Chin. Med. Res. 32, 34–35. doi: 10.3969/j.issn.1001-6910.2019.08.14

[ref27] Wang W. Hou Y. J. Wang L. Yan X. Y. Li Y. Q. Sun L. Q. . (2024). Research progress on animal model establishment and evaluation of TCM syndromes of gastric ulcer. Acta Laboratorium Animalis Scientia Sinica. 31, 1351–1360. doi: 10.3969/j.issn.1005-4847.2023.10.014

[ref28] Wang C. Jiang S. Y. Zheng H. Y. An Y. M. Zheng W. X. Zhang J. Q. . (2024). Integration of gut microbiome and serum metabolome revealed the effect of Qing-Wei-Zhi-Tong micro-pills on gastric ulcer in rats. J. Ethnopharmacol. 319:117294. doi: 10.1016/j.jep.2023.117294, PMID: 37839771

[ref29] Wang N. Tan H. Y. Li L. Yuen M. F. Feng Y. B. (2015). Berberine and coptidis rhizoma as potential anticancer agents: recent updates and future perspectives. J. Ethnopharmacol. 176, 35–48. doi: 10.1016/j.jep.2015.10.028, PMID: 26494507

[ref30] Wang S. Zhang T. Li D. M. Cao X. Y. (2025). The global, regional and national burden of peptic ulcer disease attributable to smoking from 1990 to 2021: a population-based study. Prev. Med. Rep. 51:103019. doi: 10.1016/j.pmedr.2025.103019, PMID: 40092912 PMC11908546

[ref31] Wu Y. Alomeir N. Li T. Falsettaet M. L. Yang R. Liu Y. . (2025). Effect of L. plantarum on caries prevention and the oral-gut microbiome in vivo. J. Dent. Res. 104, 993–1002. doi: 10.1177/00220345251325807 40103015 PMC13474046

[ref32] Xia Z. J. Li Y. T. Liu X. L. Mei Z. G. Zhao X. Fang K. G. . (2021). Mechanism of Huai Jiang Fang against ulcerative colitis injury via regulating NLRP3/Caspase-1 pathway. Chin. Tradit. Herb. Drug 52, 7221–7228. doi: 10.7501/j.issn.0253-2670.2021.23.016

[ref33] Yan Y. P. Chen Y. Z. Li Q. Chen B. Y. Fan Z. L. Chen S. . (2024). Effects of Rosa roxburghii Radix on ulcerative colitis in rats based on pyroptosis and neutrophil extracellular traps. Chin. Trad. Patenet Med. 46, 780–788. doi: 10.3969/j.issn.1001-1528.2024.03.011

[ref34] Yang Y. N. Cao S. J. Xu W. Y. Zang C. C. Zhang F. Xie Y. . (2022). Dual modulation of gut bacteria and fungi manifests the gut-based anti hyperlipidemic effect of Coptidis Rhizoma. Biomed. Pharmacother. 153, 1135–1142. doi: 10.1016/j.biopha.2022.113542 36076619

[ref35] Yang X. J. Cao J. M. Zhang W. Z. Wang Y. (2013). Evaluation of susceptibility testing for Corynebacterium and investigation of resistant mechanisms to quinolones. Chin. J. Nosocomiol. 23, 1235–1238.

[ref36] Yang H. Zhang M. Li H. Huang Z. J. Sun Y. Y. Li W. B. . (2024). Prevalence of common upper gastrointestinal diseases in Chinese adults aged 18-64 years. Sci Bull 69, 3889–3898. doi: 10.1016/j.scib.2024.07.048, PMID: 39562185

[ref37] Yu Z. Du Y. Cheng Y. Wu P. Gao X. (2023). Study on the correlation between intestinal flora and gastrointestinal inflammation and gastrointestinal early cancer. Chen J Clin Pharmaco 39, 3575–3579. doi: 10.13699/j.cnki.1001-6821.2023.24.007

[ref38] Zhang X. Li Q. Xia S. Y. He Y. Liu Y. Q. Yang J. L. . (2024). Proton pump inhibitors and oral-gut microbiota: from mechanism to clinical significance. Biomedicine 12:2271. doi: 10.3390/biomedicines12102271 PMC11504961 39457584

[ref39] Zhou H. X. Chen M. Qin H. W. Chen W. Da W. Zhu J. S. . (2024). Clinical observation of Qingre Huashi granules in the treatment of ulcerative colitis with damp-heat syndrome: a randomized, prospective, double-blind clinical trial. Chin J Integr Tradit West Med Dig. 32, 906–909. doi: 10.3969/j.issn.1671-038X.2024.10.14

[ref40] Zhu Y. Q. Chen B. R. Zhang X. Y. Akbar M. T. Wu T. Zhang Y. Y. . (2024). Exploration of the Muribaculaceae family in the gut microbiota: diversity, metabolism, and function. Nutrients 16:2660. doi: 10.3390/nu16162660, PMID: 39203797 PMC11356848

